# Map of the neuronal O-glycoproteome reveals driver functions in the regulated secretory pathway

**DOI:** 10.1016/j.jbc.2025.110313

**Published:** 2025-05-29

**Authors:** Thomas D. Madsen, Asli B. Topaktas, Leo A. Dworkin, John Hintze, Lasse H. Hansen, Mahnaz Nikpour, Jarkko J. Lackman, Christoffer K. Goth, Göran Larson, Emily J. Shiplett, Andrew C. Edmondson, Zhaolan Zhou, Rebecca L. Miller, Hiren J. Joshi, Sergey Y. Vakhrushev, Katrine T. Schjoldager

**Affiliations:** 1 Copenhagen Center for Glycomics, Department of Cellular and Molecular Medicine, University of Copenhagen, Copenhagen N, Denmark; 2 Department of Clinical Chemistry, Sahlgrenska University Hospital, Gothenburg, Sweden; 3 Department of Laboratory Medicine, Institute of Biomedicine, University of Gothenburg, Gothenburg, Sweden; 4 Division of Human Genetics, Department of Pediatrics, The Children's Hospital of Philadelphia, Philadelphia, Pennsylvania, USA; 5 Department of Genetics and the Epigenetics Institute, University of Pennsylvania, Philadelphia, Pennsylvania, USA

**Keywords:** granin, glycosylation, glycosaminoglycans, dense core granules (DCG), perineuronal net (PNN), mucin, central nervous system (CNS), neuron, neuroplasticity, neurotransmitter

## Abstract

Impairments in protein glycosylation, including O-GalNAc-type glycosylation, are linked to severe developmental disorders with prominent neurological involvement. However, the role of this glycosylation pathway at a cellular level is not yet fully understood. Here, we report a comprehensive map of GalNAc-type O-glycoproteins (>800) and O-glycosites (>4000) from neuronal tissues and cell lines and identify abundant O-glycosites within major classes of proteins involved in neuroplasticity, including axon guidance, membrane remodeling, and regulated vesicular secretion. Applying the map, we demonstrate that the regulated secretory pathway constitutes highly O-glycosylated proteins including Chromogranin A, a key player in dense core granulogenesis, and that correct O-glycosylation is important for its multimerization. Concurrently, genetically engineered neuronal cell lines deficient in O-glycosylation exhibit altered capacity for storing neurotransmitter noradrenaline and present enlarged neurotransmitter-containing dense core granules. Collectively, this map provides the foundation for uncovering critical roles for O-glycosylation in regulating neuroplasticity and provides evidence that dense core granule content is regulated by this pathway. Subjects: Granin, glycosylation, glycosaminoglycans, dense core granules (DCG), perineuronal net (PNN), mucin, central nervous system (CNS), neuron, neuroplasticity, neurotransmitter.

Glycosylation is the most abundant and diverse type of posttranslational modification (PTM) of proteins, and glycans serve a plethora of roles in fine-tuning protein functions and serving as ligands in important interactions (1, 2). We are likely still at infancy in elucidating and understanding the full scale of how glycans tune biological functions of proteins, but recent years have uncovered highly specific roles of, in particular, GalNAc-type O-glycans in fundamental processes, such as limited proteolytic processing of neuropeptides and other proteins (3, 4, 5, 6, 7, 8), modulation of ligand-receptor interactions (9, 10, 11, 12, 13, 14, 15, 16), and providing directional cues for proteins during trafficking (17, 18, 19). A prerequisite for the discovery of these functions was detailed knowledge of O-glycoproteomes and the positions of O-glycans.

Exploration of functions of the abundant GalNAc-type O-glycosylation (hereafter simply O-glycosylation), predicted to occur on more than 85% of all secretory proteins and differentially regulated in cells (1), has been hampered by both analytical challenges (20) and a lack of *in silico* prediction methods (21). However, recent progress in O-glycoproteomics (22, 23, 24, 25) is enabling proteome-wide deep discovery of O-glycoproteins, and we and others have employed enrichment strategies often combined with genetic engineering of cell lines to simplify O-glycan structures to generate extensive O-glycoproteome data (13, 14, 26, 27, 28, 29, 30, 31, 32, 33, 34). The current knowledge of the human O-glycoproteome is assembled in the GlycoDomainViewer (www.glyco.me) (35).

Deficiencies in genes directing O-glycosylation are emerging as a prominent group among human congenital disorders of glycosylation (CDG) (36), and these often involve severe multisystemic disorders with wide neurodevelopmental consequences (37). More distinct and subtle phenotypic outcomes may be found in CDG caused by deficiencies in genes encoding for isoenzyme glycosyltransferases with partial redundancy in functions (33, 38, 39). In this respect, O-glycosylation is exceptionally well covered by genetic and functional redundancy in the first initiation step, where a large family of 20 polypeptide GalNAc-transferase isoenzymes (40) (GALNTs) determines where O-GalNAc glycans are attached to proteins. The extent of this redundancy was clear already in early studies when mouse models with genetic ablation of *Galnt* genes did not result in obvious phenotypes (41). However, later studies demonstrated distinct subtle phenotypes for different isoenzyme deficiencies, including compromised vascular and humoral immunity (42), dyslipidemia (27), bone calcification (43), and kidney dysfunction (12). The first described human CDG caused by deficiency in a *GALNT* gene (*GALNT3*) also caused a distinct phenotype associated with hyperphosphatemia and ectopic calcifications (Familial Tumoral Calcinosis – FTC, GALNT3-CDG) (39). However, our recent study of a novel CDG caused by deficiency in *GALNT2* is showing more severe and multisystemic phenotypes with neurodevelopmental and intellectual impairments. Thus, deficiency in *GALNT2* is characterized by global developmental delay, intellectual disability, behavioral abnormalities, epilepsy, autistic features, chronic insomnia, decreased stature, white matter lesions in brain magnetic resonance scans, and dyslipidemia (33), phenotypes that are partially recapitulated in a pan-neuronal *Galnt2* knock-out mouse (44). We identified non-redundant functions of GALNT2 related to lipoprotein metabolism (27), but the role of this isoenzyme related to neurogenesis and brain function is still not known. Recently, we identified deficiencies in GALNT11 in two individuals presenting with severe neurodevelopmental challenges (manuscript *in prep*). GALNT11 non-redundantly glycosylates the low-density lipoprotein receptor (LDLR) and related receptors (LRPs) (16, 45) and is required for LRP2 and kidney function (12), but current knowledge of functions in neurogenesis is absent. While complete deficiency of *GALNT* genes is rare (38), these are prevalent GWAS (genome-wide association studies) candidate genes (1), often for neurological traits such as schizophrenia (46, 47), melatonin secretion (48) and Parkinson’s disease (49, 50).

Despite the prevalence of neurological phenotypes associated with impaired O-glycosylation, knowledge of O-glycosylation of neuronal proteins is limited and primarily sourced from large glycoproteomic studies of genetically modified cell lines of non-neuronal origin (28, 29, 30, 31, 32, 51). To meet this void, we employed a broad bottom-up glycoproteomics and informatics strategy and present detailed mapping of O-glycosites on neuronal proteins derived from human glandular, porcine, and/or rodent neuronal tissues and cerebrospinal fluid (CSF), as well as commonly used neuronal cell lines, available as a web-based resource. We identify specific sites of O-glycosylation on proteins involved in perineuronal nets, axonal guidance, synapse integrity, endomembrane trafficking, and neurotransmitter storage and release. We show that the neurotransmitter-containing dense core granules (DCGs) are comprised of highly O-glycosylated proteins including the secretogranins and demonstrate that elaborate O-glycans as well as glycosaminoglycans (GAGs) regulate the formation and size of DCGs and their capacity for neurotransmitter storage.

## Results

### Expanding the neuronal O-glycoproteome

Over the last decade, we have optimized a workflow for sensitive O-glycoproteomics employing lectin weak affinity chromatography (LWAC) for enrichment of glycopeptides followed by LC-MS/MS (23, 32, 52), and developed a large public database of the human O-glycoproteome (www.glyco.me). A major challenge for glycoproteomics is access to cell and tissue sources that express glycoproteins with highly restricted expression patterns (53), and we have identified proteins only expressed in neuronal cells that are selectively under-represented in current O-glycoproteomics data. We therefore initiated a broader program to procure neuronal tissues and cell lines, and due to challenges with human sources, we included mouse, rat, and porcine tissues.

**Figure 1:**
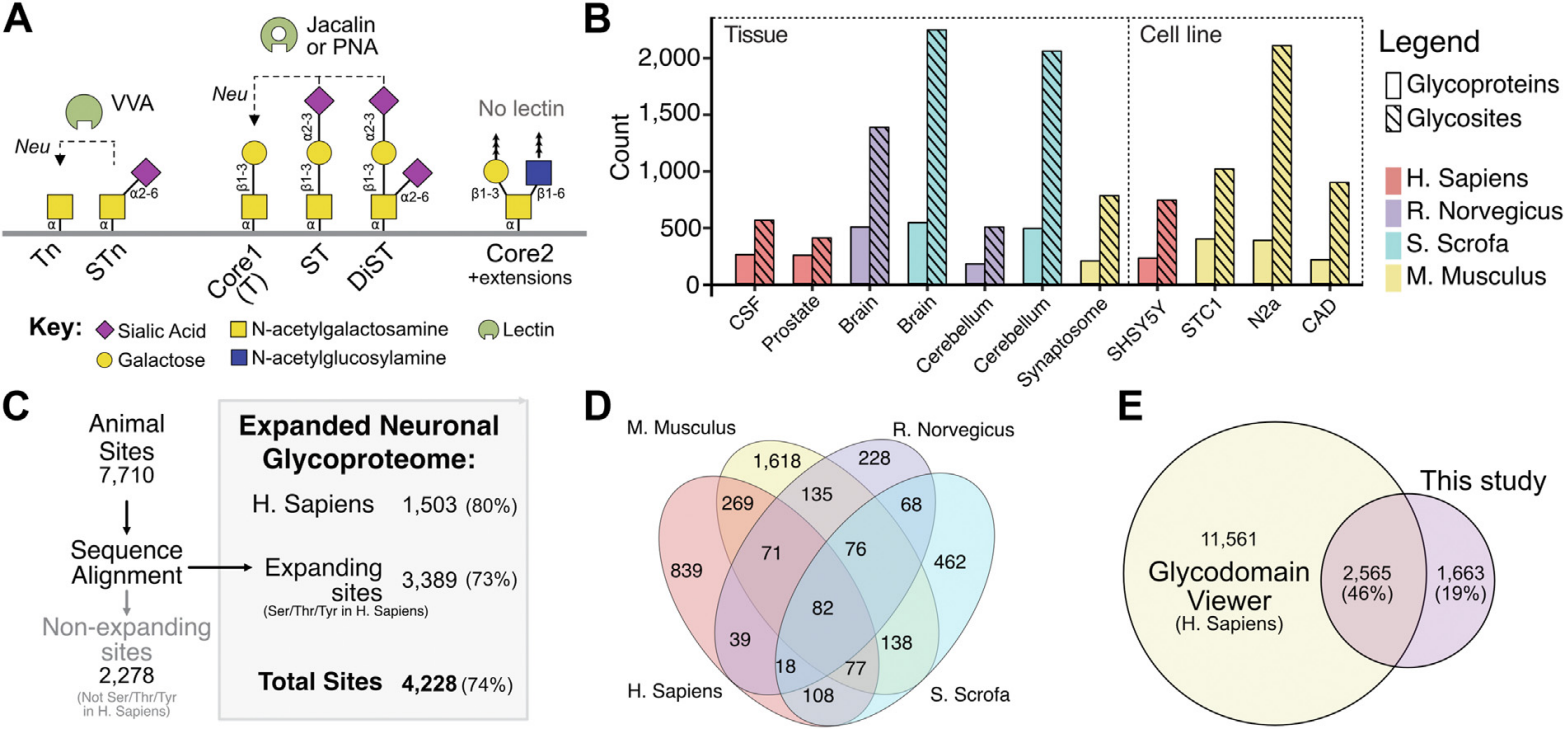
**Overview of the study of GalNAc-type O-glycosylation in neuronal tissues.***A*, schematic of common O-glycan structures found in the mammalian brain (54). The most abundant di-sialyl-T (DiST) structure is biosynthesized from the underlying Core one structure. Only a minor fraction of the Core two structure was reported. A combination of desialylation (Neu) and Core 1- (Jacalin and Peanut agglutinin, PNA) or Tn- (*Vicia villosa*, VVA) binding lectins was used to enrich for tryptic glycopeptides subjected to our glycoproteomic workflow (Sfig 1A). *B*, Bar graph displaying the number of glycoproteins and glycosites identified from individual biosources across species. *C*, overview of the expanded neuronal glycoproteome built by consolidating human O-glycosites with animal sites that are conserved in human proteins by orthologue sequence alignment (3389 sites). Sequence alignment was performed using ClustalO on the full-length sequence of each animal glycoprotein and its human orthologue. Glycosites that aligned to Ser/Thr/Tyr were included in the expanded neuronal glycoproteome (4228) (*bottom*). Proportion of human sites, expanding sites and total number of sites predicted by the Net-O-Glyc 4.0 algorithm are stated in parentheses. *D*, species source overlap among the total 4228 O-glycosites of the expanded dataset. *E*, the expanded neuronal O-glycoproteome contributes novel O-glycosites compared to the current aggregate of experimentally identified human O-glycosites (*yellow*) (https://glyco.me/docs/resources/glycodomain/). The proportion of sites that are experimentally verified in human samples is given in parentheses.

O-glycoproteomic studies were performed on neuronal tissues and cell lines employing LWAC for enrichment of O-glycopeptides (GalNAc-type) followed by LC-MS/MS (14). Human (prostate and CSF) as well as non-human neuronal tissues (rat and pig whole brain and cerebellum) and murine cell lines STC-1 and N2a samples were previously reported (14), however, the glycoproteomics data were only extracted and analyzed for neuropeptides (∼10% of all peptide-spectrum matches (PSMs)). Here, for the first time, we analyze the entire neuronal tissue datasets and include data from murine synaptosomes as well as SHSY-5Y and CAD neuronal cell lines. For O-glycoproteomics, all samples (tissues, fluids, and cell lines) were extracted for proteins and subjected to proteolytic digestion and LWAC (Fig. S1*A*). The vast majority of O-glycans found in CSF and in whole-brain lysates are based on Core1 O-glycans (54). Thus, to capture neuronal O-glycopeptides for our bottom-up LC-MS/MS approach, we pretreated the samples with neuraminidase to desialylate the Core1 structures and enriched glycopeptides using the Core1-recognizing Peanut Agglutinin (PNA) and/or Jacalin lectins in the LWAC enrichment (Fig. 1*A* and Table S1). For cell lines naturally deficient in O-glycan elongation (mouse N2a *Cosmc*^−/−^) that produce homogeneous Tn/STn O-glycans, we used Vicia Villosa Agglutinin (VVA) lectin instead. We identified between 180 and 550 O-glycoproteins from each source with a total of >2800 O-glycoproteins (Fig. 1*B*). In total of ∼122,000 PSMs were identified (Fig. S1*B* and Table S2) describing 9213 native glycosites (7710 animal + 1503 human) (Table S3).

Since one part of the identified glycosites was obtained from non-human species, we used orthologous conservation analysis of 7710 identified animal glycosites (Clustal-O sequence alignment) to expand the O-glycoproteomic dataset for humans (Fig. 1*C*). A total of 5432 (70.5%) rodent or porcine glycosites aligned to 3389 Ser/Thr/Tyr residues in orthologous human proteins (Table S4 and Fig. S1*C*). The residual 2278 animal O-glycosites that did not align to a Ser/Thr/Tyr residue were considered non-conserved, species-specific O-glycosites and were excluded from the dataset. Of the 1503 identified human sites, 664 (269 + 71 + 82 + 77 + 39 + 18 + 108) overlapped with the conserved animal sites (Fig. 1*D*). Collectively, this amounts to 4228 O-glycosites of which 1503 were experimentally identified in human biosources, and 2725 were experimentally identified in animal biosources and conserved in the orthologous human protein (Table S4). We refer to this as the expanded dataset in the following analyses. An analysis of the expanded dataset by the NetOglyc4.0 algorithm (32) predicted 73% of the sites to be glycosylated in the human sequence. This prediction rate was similar to that for the experimentally identified human O-glycosites alone (80%) (Fig. 1*C*), thus supporting our approach for expansion of the dataset. Comparing the expanded dataset with the largest compilation of O-glycosites (glyco.me) revealed an overlap as well as a distinct expansion with 1663 novel sites (Fig. 1*E*). The glyco.me database is built mostly from cell line data, and this increase demonstrates the importance of including native tissues.

### The neuronal tissue O-glycoproteome

**Figure 2:**
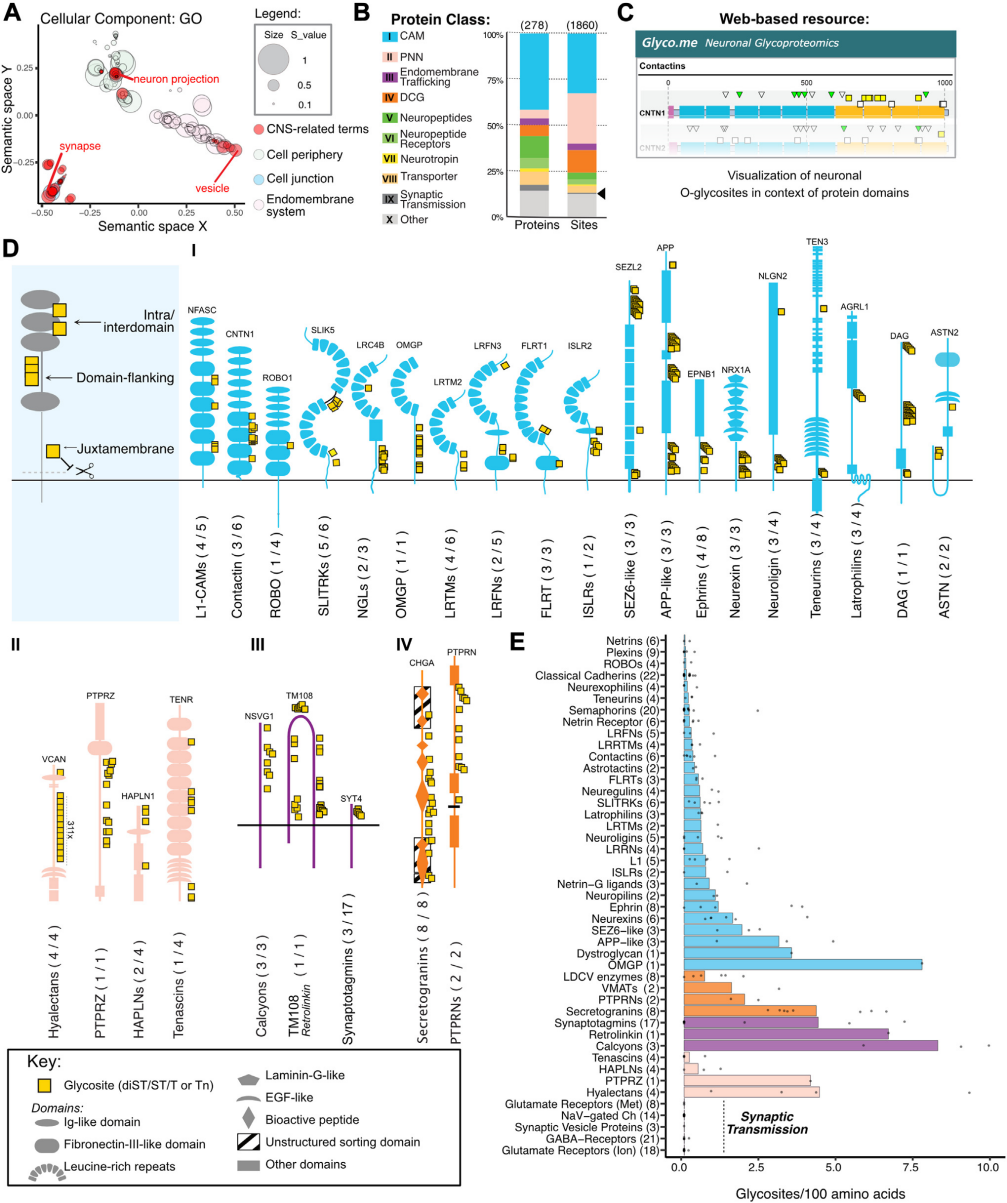
**Graphic representation of the expanded neuronal tissue O-glycoproteome.***A*, cellular component GO-term analysis of the expanded neuronal O-glycoproteome presented as a reduced dimensionality plot using ago-tool (122). Similar GO-terms are grouped based on similarity of proteins in associated parent-terms identified using the rrvgo algorithm (123). Terms related to central nervous system functions are highlighted in *red*, with the most significant term annotated. *B*, distribution of glycoproteins and glycosites across the 10 major classes of neuronal protein families (I-X) in the expanded O-glycoproteome. The arrowhead highlights the (relatively) few O-glycosites identified in proteins associated with synaptic transmission and/or action potential propagation. *C*, screenshot from a web-based resource with schematics of 1332 identified O-glycosites visualized in the context of local folds/domains and other PTMs in 283 proteins (https://neuronal.glycomics.ku.dk/). Proteins are divided into 48 families based on structure-function relationships to facilitate the identification of general trends of O-glycosites between family members. Families are retrieved from the HGNC database (https://www.genenames.org/) and, in a few cases, from literature reviews. *D*, schematic depiction of O-glycosites in relation to protein domains within a representative member of highlighted families. Family name is stated below each schematic with the number of proteins carrying O-glycosites (x) out of the total number of family members (y) stated in parentheses (x/y). *E*, Bar graph showing glycosite density within the selected families. Bar height represents the average density of O-glycosites between family members. Dots represent the number of glycosites per 100 extracellular amino acids in each individual protein. Total number of members per family is annotated in parentheses. CAM, cell adhesion molecule; DCG, dense core granule; PNN, perineuronal net.

Analysis of the expanded O-glycoproteome dataset by gene ontology (GO) and plotting enriched GO terms in a semantic similarity scatterplot revealed significant enrichment of terms related to synapse, neuron projection and vesicles (Fig. 2*A* and Table S5). Guided by this, we manually curated the expanded dataset and found that the bulk of glycosites with potential relevance for neuronal processes (1860 out of 4228 sites, Fig. S1*D*) was found in proteins controlling processes, such as cell–cell adhesion, dense core granules (DCG) granulogenesis, endomembrane trafficking, perineuronal net formation (PNN), neuropeptide and receptor signaling, and all general processes involved in neuroplasticity (55)) (Fig. 2*B*). To appreciate conserved glycosylation patterns within protein families, we further selected 54 families carrying the majority of glycosites in neuronal proteins and depicted the identified glycosites in the context of protein domain structures and other PTMs. These results are compiled in a web-based resource to facilitate communal investigation of the neuronal O-glycoproteome (https://neuronal.glycomics.ku.dk) (Fig. 2*C*).

Surveying the web-based resource and proteins from the top four most glycosylated classes (CAM, PNN, Endomembrane trafficking and DCGs) revealed that O-glycosites are relatively equally distributed in three main local structural contexts: (1) intradomain, (2) domain flanking, and (3) juxtamembrane (Fig. 2*D*). Interestingly, few or no O-glycosites were located in protein families directly involved in action potential propagation such as GABA and glutamate receptor subunits, voltage-gated ion channels or proteins of the small clear core vesicles such as the neurotransmitter-concentrating synaptic vesicle two glycoproteins (Fig. 2, *B* and *E*), despite the fact that these proteins are highly expressed in the analyzed tissues (53), often found N-glycosylated (56, 57, 58), and known to traffic the secretory pathway. In support of this observation, NetOGlyc4.0 predicted few O-glycosites in the extracellular domains of these proteins (Fig. S1*E*). Thus, our map suggests that the bulk of O-glycosylation in neuronal proteins is likely to be involved in regulating neuroplasticity and synaptic integrity in specific aspects, as discussed in the following, rather than directly regulating fast synaptic transmission and propagation of action potentials.

#### Neuronal cell adhesion molecules

Cell adhesion molecules (CAMs) comprise a large class of proteins that tether neuron-neuron and neuron-glia interactions and/or are involved in axonal guidance (59). We found O-glycosites in several CAM families (Fig. 2, *D-I*). In the Ig-superfamily members like contactins, roundabout proteins (ROBOs), and L1-CAMs, we identified intradomain and interdomain glycosites, in or between fibronectin-III- and Ig-like-domains. In leucine-rich repeat-, SEZ6-, ephrin-, and amyloid polypeptide (APP)-like proteins, clusters of O-glycosites in domain-flanking linkers likely function to modulate flexibility and extension of domains in these CAMs. O-glycosites in juxtamembrane regions are also found in APP-like, neurexins, neuroligins, and teneurins, where they may serve to protrude ectodomains from the cell membrane, as found for LDLR (60). O-glycosites in the juxtamembrane region are also known to co-regulate ectodomain shedding by inhibiting limited proteolysis by metalloproteases, which was previously demonstrated for APP and neurexin-1 (61).

O-glycosites were frequently identified within folded domains (intra-domain), *e.g.*, in Ig-like, Fibronectin-III-like, leucine-rich repeats, EGF-like, and Laminin-G-like domains. We identified numerous O-glycosites within FN3-type domains, a 100 amino acid β-sandwich structure widely found in extracellular proteins. These O-GalNAc glycosites were primarily located in the connecting loop between the B and C strands, and specifically on the E and G strands (data not shown). The latter may resemble how O-mannose (another O-linked glycosylation type) glycosites are found in the B and G strands of Cadherin-like domains (62), and although the specific functions of these O-mannose glycans are still to be explored, deficiencies in the isoenzymes directing O-mannosylation of cadherins and protocadherins result in severe developmental defects in the brain (63). FN3 domains in NRCAM are pivotal for interaction with integrins and other CAMs and NRCAM variants in neurodevelopmental syndrome patients cluster in the third FN3 domain, suggesting an important involvement in NRCAM structure and function where glycans may play a regulatory role (64).

#### Perineuronal nets

Strikingly, almost a quarter of all identified O-glycosites in the expanded neuronal glycoproteome were found within just five chondroitin sulfate proteoglycans (CSPGs): Phosphacan (PTPRZ) and the four members of the hyalectan family (aggrecan, neurocan, brevican, and versican) (Fig. 2, *D*-*II*). These CSPGs are the major constituents of the special extracellular matrix known as perineuronal nets that exist in the pericellular space between neurons and astrocytes and function by crosslinking hyaluronic acid in a meshwork between adjacent cells (65, 66). The O-glycans were positioned in the central unstructured domain of the CSPGs and resemble densely glycosylated mucin-like domains. Only aggrecan of the CSPGs displays a variable number tandem repeat structure typically associated with mucins (13–33× PGVEDISGLPSGEVLETAA, depending on isoform (67)), whereas the others are devoid of such a tandem repeat structure, and their unstructured domains are more akin to the mucin-like domain in syndecan-3 (68). Among other components of the perineuronal nets, we also identified dense mucin-like distribution of O-glycosites in phosphacan and more limited O-glycosites in Tenascin-R/C, as well as the hyaluronan and proteoglycan link proteins HAPLN1/2, suggesting widespread roles for O-glycosylation in the regulation of cell-specific PNN functions.

#### Endomembrane trafficking

Synaptotagmins are crucial players in the regulated secretion of vesicular content (69). It was previously suggested that O-glycosylation in the N-terminal luminal domain of the vesicle-fusion protein synaptotagmin-1 determines subcellular localization in cell lines (17). We identified similar N-terminal luminal glycosites in 3 (1, 4, 5) out of the 17 known synaptotagmins, suggesting that O-glycosylation may regulate the subcellular trafficking of other members of this protein family (Fig. 2*D*-*III*). Among other proteins involved in endomembrane trafficking, we identified similar luminal O-glycosites in NSG1-3 (Neuronal vesicle trafficking associated proteins) as well as in retrolinkin (TMEM108). These proteins are involved in the trafficking of important synaptic proteins, such as neurotransmitter receptors and CAMs, and deficiency within these proteins is associated with both Alzheimer’s disease and schizophrenia (70, 71), although the underlying mechanisms and potential roles of O-glycosylation are poorly understood.

#### Regulated vesicular secretion

While proteins of the small clear core vesicles involved in synaptic transmission appeared to contain relatively few O-glycosites, we found widespread O-glycosites on proteins of DCGs (Fig. 2*D*-*IV*). DCGs constitute the main storage compartment for neurotransmitters such as bioactive peptides and biogenic amines (noradrenaline, serotonin, and dopamine) in neurons and endocrine cells that undergo regulated secretion in response to specific stimuli. The bulk of glycosites within this compartment was found in the luminal domains of transmembrane neurosecretory protein families PTPRNs (ICA512 and Phogrin) and in the soluble secretogranins, which are important for the cargo selection and biogenesis of DCGs (72, 73, 74, 75). We further identified isolated O-glycosites in DCG cargo proteins, including neuropeptide-processing enzymes (PAM, PCSK1, CBPD, and QPCT) as well as neuropeptides (and the related endocrine peptide hormones). Site-specific glycosylation of neuropeptides is known to regulate their half-lives and receptor activity (13, 14, 76). Of note, we did not identify O-glycosites on the widely expressed neuropeptide processing enzyme carboxypeptidase E, in agreement with only a few sites predicted by NetOGlyc4.0.

### Probing the DCG-related regulated secretome

**Figure 3:**
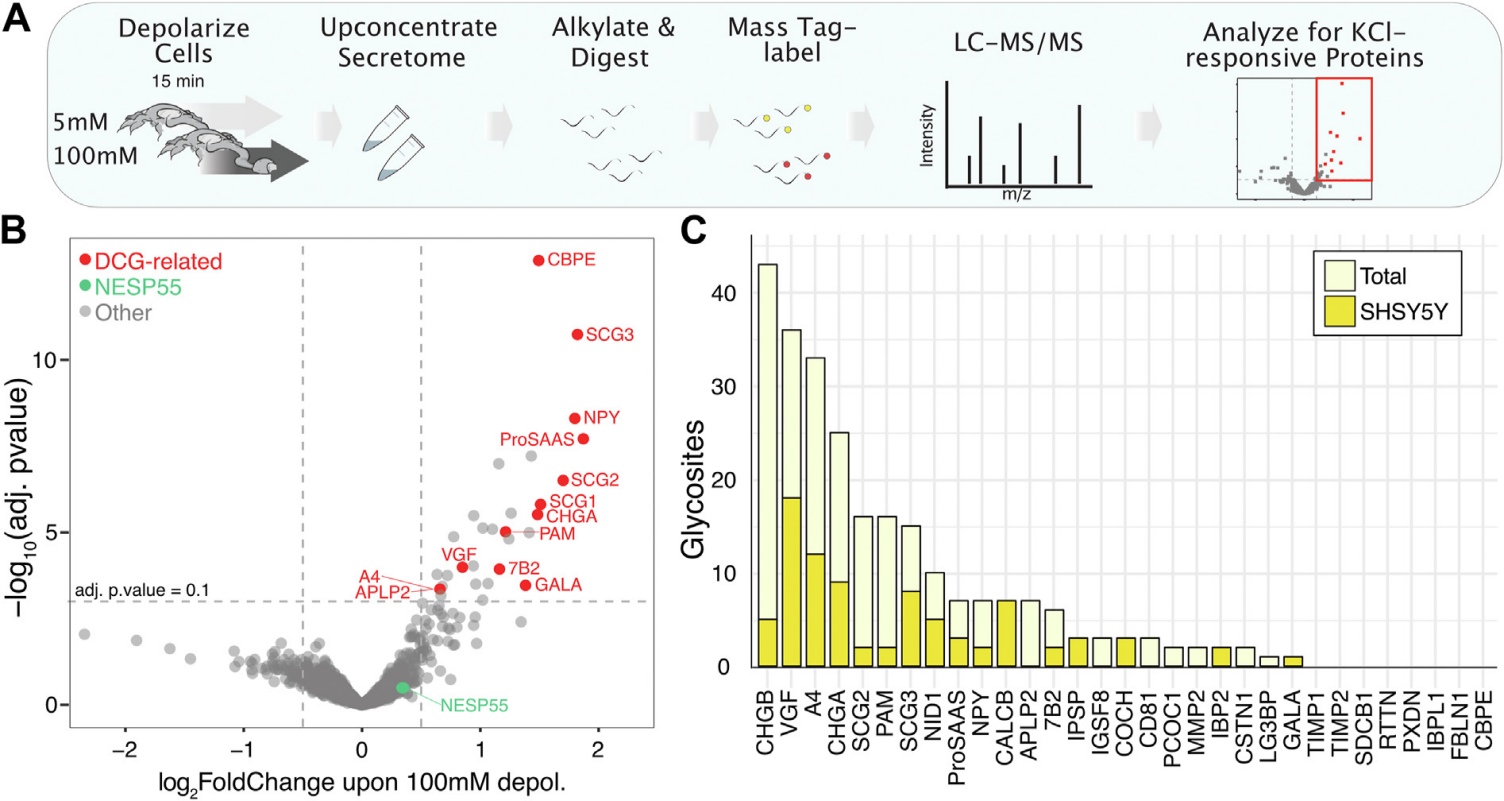
**Probing the DCG regulated secretome in human SH-SY5Y cells.***A*, schematic workflow with/out KCl depolarization, concentration by protein precipitation, digestion, differential TMT-labelling, and LC-MS/MS tandem-mass-tag based detection. The secretomes of five individual clones (n = 5) per condition were analyzed as described in the methods section. *B*, Volcano plot showing log_2_ fold change of proteins secreted after depolarization with significantly (adjusted *p*-value >0.1) DCG-related proteins highlighted in *red*. *C*, stacked bar plot of glycoproteomics data from the expanded dataset (total) and from SH-SY5Y cells showing the total number of O-glycosites found on proteins undergoing regulated secretion.

The abundance of O-glycosites identified on DCG proteins suggested important roles in neurotransmitter packaging and signaling and prompted us to explore potential functional roles of O-glycans in the regulated secretion of soluble cargo. We selected the catecholaminergic neuroblastoma cell line SH-SY5Y (77) and the neuroendocrine gut-peptide hormone-producing STC-1 cells (78) as models known to produce DCGs. To qualify the DCG content in these cell models, we developed a multiplex proteomics assay for KCl-stimulated secretion by the SH-SY5Y and STC-1 cells (Fig. 3*A*). Comparing the protein identifications of regulated secretion (100 mM KCl) to basal secretion (5 mM KCl) resulted in 31 (SH-SY5Y) and 84 (STC-1) differentially secreted proteins (Figs. 3*B* and S2*A*, Tables S6, and S7). Among the most KCl-responsive proteins, we detected well-known DCG-related resident proteins, including seven out of eight members of the secretogranin family (CHGA, CHGB, 7B2, SCG2, SCG3, SAAS, and VGF), as well as carboxypeptidase E and PAM in both cell lines. The eighth secretogranin member, NESP55, is specifically known to follow the constitutive secretory pathway (79) and was not undergoing regulated secretion in our analysis, illustrating the power of our assay to distinguish regulated from constitutive secretion. Neuropeptides such as NPY and Galanin (SH-SY5Y), as well as GIP and SCT (STC-1), also followed the regulated secretory pathway, but they were detected in a cell-specific manner in agreement with their expression (78, 80). To the best of our knowledge, this is the first method described that quantifies the soluble secretome from depolarized cells in culture, and it has broad applications for studying regulated secretion.

To explore the abundance of O-glycoproteins in the regulated secretomes, we applied our extended neuronal glycoproteome map including the cell line specific O-glycoproteome and found that 28 out of 31 top-responsive proteins in the SH-SY5Y cell line (Fig. 3*C*) and 40 out of 84 proteins in the STC-1 cell line are O-glycoproteins (Fig. S2*B*). The number of O-glycosites per protein varied with up to 44 O-glycosites identified in CHGB. Thus, the soluble cargo of DCGs is predominantly O-glycoproteins.

### Elaborate O-glycans are not required for regulated secretion

**Figure 4:**
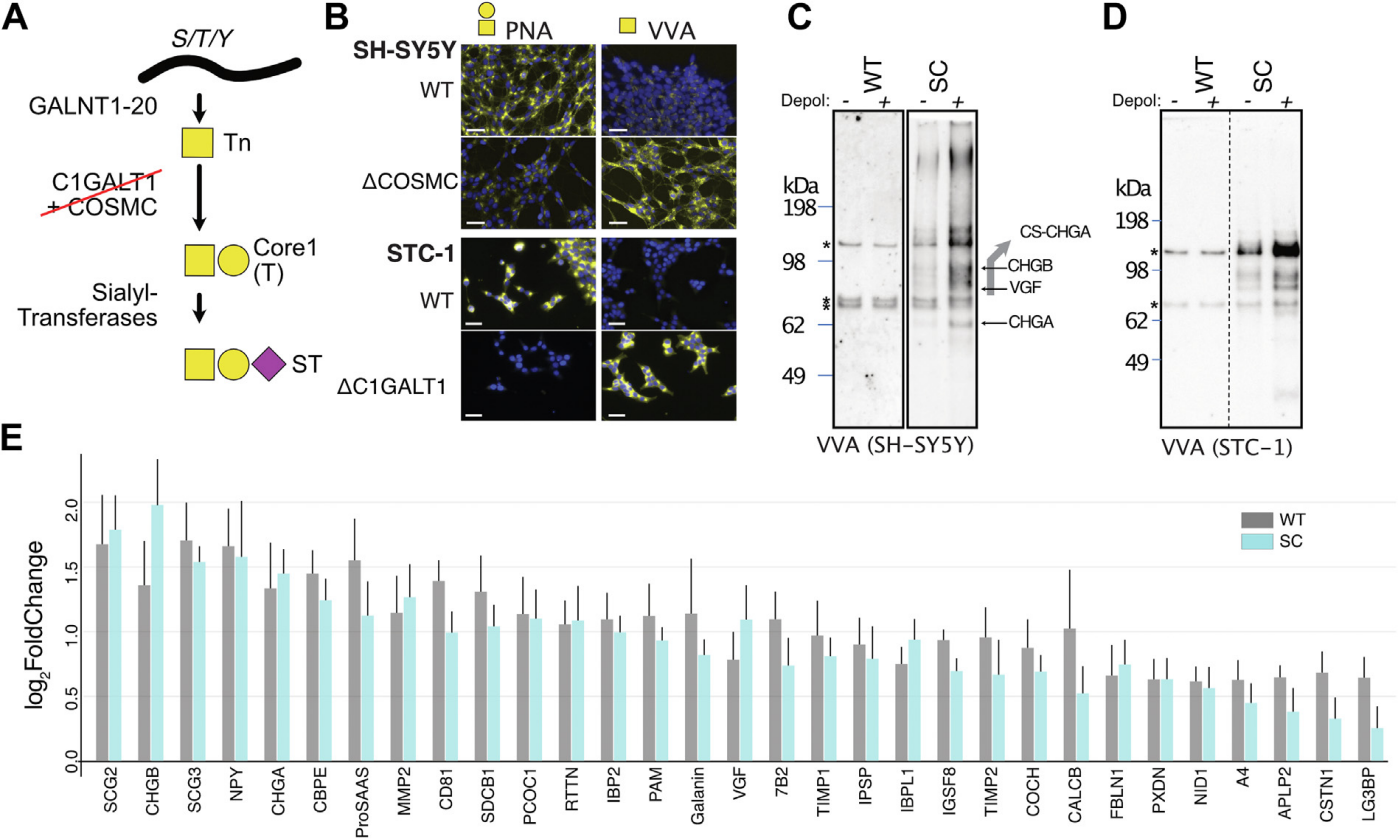
**Probing roles of elaborate O-glycans for regulated secretion.***A*, targeting C1GALT1 enzyme or its obligate private COSMC chaperone by CRISPR-Cas9 results in SC cells producing truncated (Tn) O-glycans on proteins. *B*, representative immunofluorescence images of STC-1 and SH-SY5Y WT and SC cells. Pretreatment with neuraminidase shows the gain of Tn (VVA) and loss of Core 1 (PNA) in SCs and validates the glycoengineering. *C* and *D*, Western blot analysis of secretomes ± depolarization from SH-SY5Y and STC-1 WT and SC cells. Blots were pretreated with neuraminidase and probed with the VVA lectin to selectively identify O-glycoproteins with truncated Tn O-glycans. *Arrows* indicate relative migration of highly glycosylated granins (deferred from separate western blots (Sfig 2C)). A VVA-reactive smear that co-mobilizes with both chondroitin sulfate and high molecular weight CHGA is indicated by a *grey arrow*. *Asterisks* (∗) denote background bands. *E*, Bar plot showing the mean relative secretion of the KCl-responsive proteins in SH-SY5Y^WT^ and ΔCOSMC (SH-SY5Y^SC^). Error bars represent + standard error of the mean, n = 5 clones.

Based on our discovery that the soluble content of DCGs predominantly consists of O-glycoproteins, we aimed to investigate the potential functions of O-glycans in the process of regulated secretion. As the initiation step of O-glycosylation is covered by up to 20 isoenzymes and global elimination of protein O-glycosylation in cells therefore requires vast recombinational KO engineering, we targeted *C1GALT1* or its obligate chaperone *COSMC* to limit biosynthesis of O-glycans to the initial α-GalNAc residues (also designated Tn) with the intent of disrupting functions of the native elaborated O-glycans (Fig. 4*A*). To account for biological variability, we generated five clonal isogenic cell lines of both SH-SY5Y-Δ*COSMC* (SHSY-5Y^SC^) and STC-1−Δ*C1GALT1* (STC-1^SC^) (referred to as SimpleCells SC (23)) alongside five individual WT clones of each cell line, and confirmed loss of elaborated Core-1 O-glycans (PNA) and gain of truncated Tn O-glycans (VVA) by lectin labeling (Fig. 4*B* and Table S8).

Interesting, minor labeling of residual Core1 O-glycans was observed in the SH-SY5Y^SC^, which could be explained by partial compensation from the *COSMC2* gene (Fig. S3). Previously, *COSMC2* was presumed to represent a pseudogene with 93% homology to *COSMC* (81), but here we demonstrate that knockout of *COSMC2* in HEK293^ΔCOSMC^ cells abolishes residual PNA staining (Fig. S3*C*) and that introduction of *COSMC2* in a naturally *COSMC*-null background partially rescues PNA staining in N2a (Fig. S3*E*), suggesting that the gene product can serve as a chaperone for C1GalT1 and allow extension of the Tn-antigen to T in these cells, albeit at much lower efficiency compared to COSMC.

Moreover, we could demonstrate that the SH-SY5Y cells deficient in O-glycan extension do not sialylate the Tn O-glycans to produce STn glycans (Fig. S3*F*), further eliminating any potential biological roles driven by sialylation.

One advantage of eliminating O-glycan biosynthesis beyond the first α-GalNAc is that endogenous secreted O-glycoproteins can be distinguished from serum-derived elaborated O-glycoproteins by probing with the high-affinity VVA lectin after SDS-PAGE and Western blotting. We therefore probed the secretomes from SH-SY5Y^SC^ and STC-1^SC^ cells, which revealed diffuse VVA reactive bands over a broad Mw range in the constitutive secretome, which increased in intensity upon KCl-depolarization (Fig. 4, *C* and *D*). Interestingly, the VVA banding patterns were similar for the SH-SY5Y^SC^ and STC-1^SC^ cells, suggesting that common O-glycoproteins were involved. Our glycoproteomics data showed that among the KCl-responsive proteins, members of the secretogranin family (CHGA, VGF, and CHGB) contained the most O-glycosites (Fig. 3*C*), and we confirmed this by protein-specific Western blot analysis, revealing that CHGA, VGF, and CHGB bands comigrated with the VVA-reactive bands identified in the secretome of SH-SY5Y cells (Fig. S4*A*).

We then asked whether the truncation of O-glycan structures to the single α-GalNAc monosaccharide ithe regulatedegulated secretion of individual proteins in our cell models. We compared constitutive and regulated secretomes from the isogenic cell line pairs SH-SY5Y^WT^/SH-SY5Y^SC^ and STC-1^WT^/STC-1^SC^ using TMT multiplexing and differential proteomics as described earlier. For comparative analysis, secretomes were divided into multiple 10-plex batches across the five cell clones of each genotype with and without stimulation and we combined each batch into one dataset using internal references (82). Perhaps surprisingly, the genetic truncation of O-glycosylation with elimination of elaborate O-glycans in cells did not substantially affect the regulated secretomes in either of the tested cell types (Figs. 4*E*, S4, *B*−*D*, S6, and S7). Worth noting are minor changes in secretion of the bioactive peptide precursors galanin and CALCB (SH-SY5Y) and secretin (STC-1).

In summary, we demonstrated that secretogranins are the major O-glycan carriers in the DCG-regulated secretomes and that elongated O-glycans do not influence the secretion of DCG-residing proteins.

### CHGA undergoes differential modification with O-glycans and chondroitin sulfate in cells

**Figure 5:**
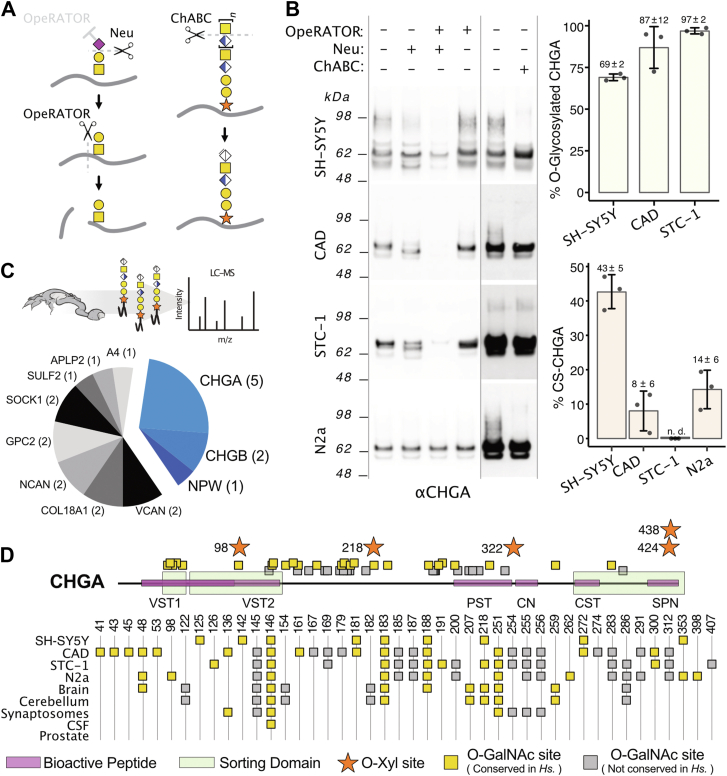
**Subsaturated levels of CS GAGs and O-glycans on CHGA.***A*, schematic depiction of enzymatic specificities used in the diagnostic validation of CHGA-glycosylation. The glycosyl hydrolases Chondroitinase ABC (ChABC) and neuraminidase (Neu) hydrolyze the chondroitin sulfate elongation and sialic acid capping, respectively. The mucinase OpeRATOR cleaves the protein backbone N-terminal to Core1 O-glycans and is inhibited by the presence of sialic acid on the Core one structure (sialyl-Core 1). *B*, Western blot detecting secreted CHGA with/without enzymatic treatments from four different cell types (SHSY-5Y, CAD, STC-1 and N2a). Collected media were pretreated with chABC, OpeRATOR and/or neuraminidase (Neu) as indicated. CHGA with truncated Tn O-glycans produced in COSMC-deficient N2a cells was unaffected by both neuraminidase and OpeRATOR treatment as expected. Note that contrast was increased for chABC assay western blots. Bar plots show the result of Western blot quantification of the fraction of mucinase-sensitive O-glycosylated CHGA (*Top*) and chABC-sensitive CS-carrying CHGA (*Bottom*) from three independent experiments. Error bars represent standard deviation. n.d.: not detected. *C*, Pie chart summary of the relative number of identified O-Xyl attachment sites identified within each proteoglycan in the SH-SY5Y^WT^ secretome. *D*, schematic summary of O-GalNAc and O-Xyl sites identified in CHGA relative to bioactive peptides and sorting domains. The table highlights the source of each O-GalNAc-site, where numbers report the glycosite position after alignment to the human sequence. *Yellow squares* represent human native or expanding sites, whereas *gray squares* represent sites that do not align to a Ser/Thr/Tyr in the human sequence during sequence alignment. CN, Chromostatin; CST, Catestatin; Hs., *Homo sapiens*; PST, Pancreastatin; SPN, Serpinin; VST, vasostatin.

Our finding of abundant O-glycans on secretogranins prompted us to determine the O-glycosylation of endogenously secreted CHGA in different WT cell lines of neuronal origin. First, we treated conditioned media from SH-SY5Y, CAD, STC-1, and N2a with neuraminidase, followed by OpeRATOR O-glycoprotease (29) (Fig. 5*A*) and analyzed the digestion products by Western blotting. Neuraminidase treatment alone induced a minor shift in migration of CHGA secreted from SH-SY5Y cells, whereas CHGA from CAD and STC-1 cells appeared to split into two distinct bands, suggesting differential O-glycosylation and/or higher degree of sialylation. The O-glycoprotease, in combination with the neuraminidase, digested the CHGA bands from CAD and STC-1 cells to near completion, suggesting close to a full occupancy of O-glycosites, while a minor CHGA band remained undigested in the SH-SY5Y cells, suggesting incomplete occupancy of O-glycosites (Fig. 5*B*).

CHGA has been reported as a proteoglycan in CSF (Noborn *et al.* 2015). Proteoglycans are proteins with long sulfated glycosaminoglycan (GAG) chains, such as chondroitin sulfate (CS) or heparan sulfate (HS), attached to select serine residues. We found approximately 50% of CHGA secreted from SH-SY5Y to be decorated with CS by analyzing the effects of chondroitinase ABC treatment on SDS-PAGE migration, where a slow migrating immunoreactive smear of CHGA was completely susceptible to digestion of CS by Western blotting (Fig. 5*B*). Interestingly, we observed substantial differences in the degree of CS glycoforms of CHGA between cell lines. Thus, CHGA secreted from STC-1 cells did not appear to carry CS at all, whereas secreted CHGA from CAD and N2a cells both contained significant CS (Fig. 5*B*). To further explore proteoglycans in the SH-SY5Y secretome, we performed dedicated GAG glycoproteomics on conditioned media from SH-SY5Y cells (83) where we identified 21 secreted proteins with GAGs attached in total (proteoglycans) (Fig. 5*C* and Table S9). We confirmed previously published CS glycosites on CHGA, CHGB, and neuropeptide W, and identified four new CS glycosites on CHGA, indicating a total of up to 5 CS sites in CHGA.

To explore these findings further in relation to regulated secretion in cells, we employed a genetic engineering strategy to truncate GAG glycosylation in SH-SY5Y cells by KO of the *B4GALT7* gene that controls the second step in the GAG biosynthesis process to generate SH-SY5Y^ΔB4GALT7^ cells that only attach the first xylose to Ser residues (84, 85) (Fig. S5*A*). We probed the regulated secretomes for CHGA and confirmed that the truncation of GAG synthesis resulted in loss of the high Mw smear (Fig. S5*B*) shown to be sensitive to chondroitinase ABC treatment. We then employed an anti-CS antibody (mAb CS-56) to demonstrate that CHGA is the main carrier of CS in the regulated secretome of SHSY-5Y cells (Fig. S5*C*). Note that CS-56 labels the slow-migrating CHGA band in Western blot analysis in SHSY-5Y^WT^ as well as the O-glycan-engineered SHSY-5Y^SC^ cells, whereas there is no binding with SH-SY5Y^ΔB4GALT7^ cells.

Together, these results demonstrate that CHGA undergoes cell-specific and variable glycosylation with O-glycans and CS, providing for potential regulatory roles of glycosylation (Fig. 5*D*).

### Glycans serve functions in granin self-aggregation and neurotransmitter storage

**Figure 6:**
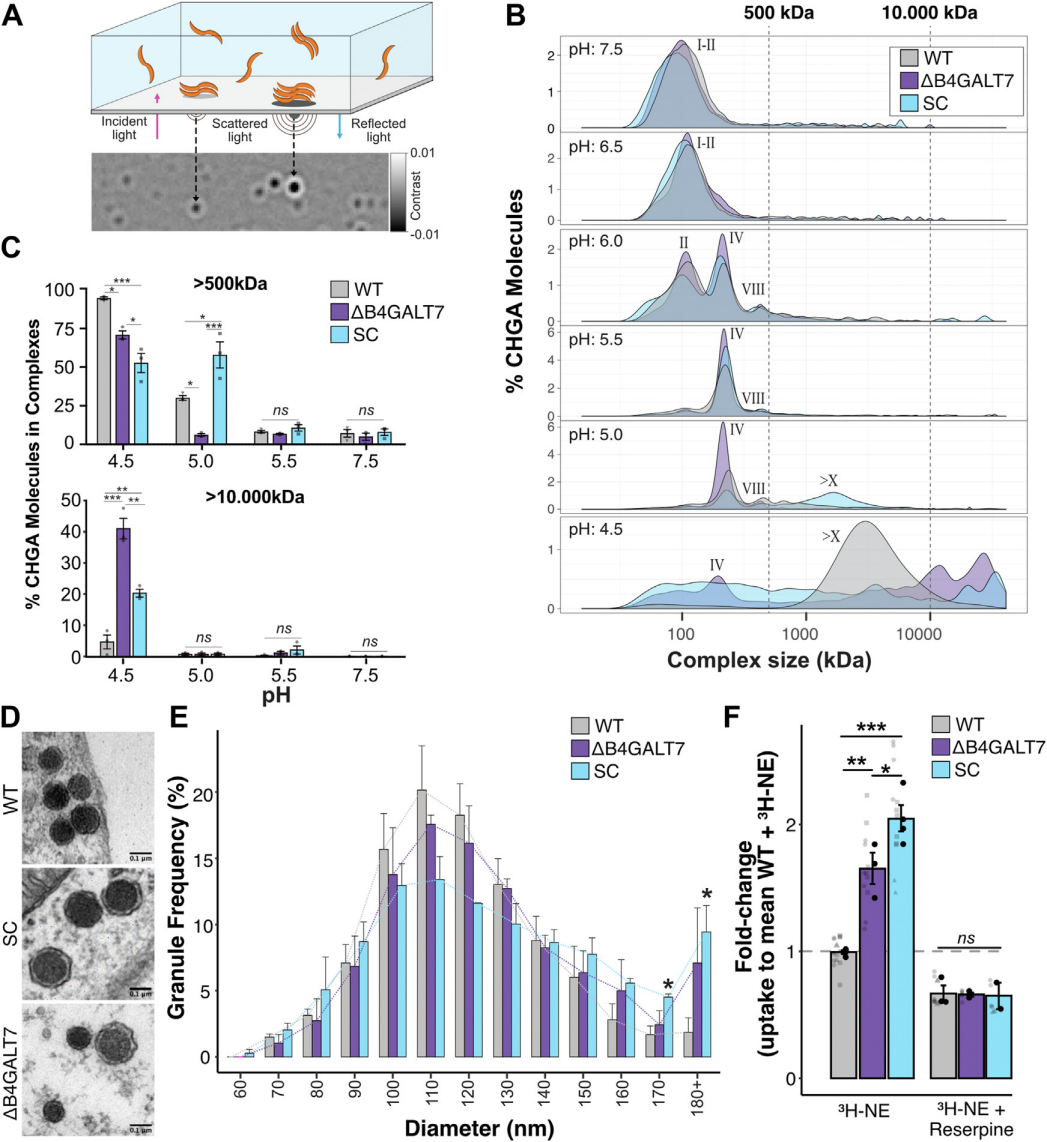
**Glycans regulate CHGA multimerization, DCG size, and DCG neurotransmitter uptak**e. *A*, schematic of mass-photometry-based analysis of purified protein complex mass. *B*, molecular distribution of CHGA in homomeric complexes formed at 200 nM concentration at different pH. Roman numbers (I, II, IV, VIII) refer to mono, di, tetra, and octameric CHGA complexes, while masses >500 kDa (X) correspond to large complexes of >10 CHGA molecules. Recombinant secreted CHGA glycoproteoforms were isolated from HEK293^WT^, HEK293^SC^, and HEK293^B4GALT7^ cells as indicated. The number of CHGA molecules in each complex (count) was calculated based on the molecular weight of CHGA (50.4 kDa, after removal of the His-tag). Raw counts are presented in Sfig 7. *C*, quantification of CHGA molecules in complexes of masses >500 kDa (*top panel*) and >10.000 kDa (*bottom panel*) at different pH. Three independent measurements, One-way ANOVA with Tukey’s *post hoc* test. *D*, representative transmission electron micrographs of SH-SY5Y^WT^, SH-SY5Y^SC^ and SH-SY5Y^ΔB4GALT7^ cells show dense core granules with a thin bright halo in all three cell lines. Scalebars = 0.1 μm. *E*, frequency distribution of binned DCG sizes from SH-SY5Y^WT^, SH-SY5Y^SC^ and SH-SY5Y^ΔB4GALT7^ cell lines. A range of 272 to 633 granules per clone for three clones was analyzed per genotype. Unpaired two-sided *t* test between WT and KO (*F*) Bar graph showing ^3^H-noradrenaline uptake in SHSY-5Y^WT^ and glycoengineered cell lines with and without inhibition of VMAT-mediated vesicle uptake by Reserpine. Two to 3 clones per genotype measured in duplicate wells from three independent experiments, one-way ANOVA with Tukey’s *post hoc* test. Error bars represent S.E.M, ns = *p* > 0.05, ∗*p* < 0.05, ∗∗*p* < 0.01, ∗∗∗*p* < 0.001.

A major role of the granins, including CHGA, is to initiate granulogenesis by self-aggregation onto the *trans-*Golgi network membranes (86). While exogenous addition of CS has been shown to modulate CHGA oligomerization (87), the role of endogenous CHGA glycoproteoforms is unexplored. We therefore investigated how recombinant CHGA isolated from glycoengineered HEK293 cells formed oligomers at different pH values using single-molecule mass photometry (Fig. 6*A*). Of note, CHGA recombinantly produced in HEK293 carries both GAGs and O-glycans at levels comparable to those of CHGA endogenously produced by SH-SY5Y cells (Fig. S6). CHGA presented as broad peaks corresponding to masses of monomers and dimers at neutral pH. Decreasing pH (0.5 increments) revealed the appearance of tetramers at pH 6, octamers at pH 5.5, and larger (megadalton >500 kDa) oligomers at pH 5 (Fig. 6*B*). Interestingly, at pH 5, CHGA with truncated O-glycans (HEK293^SC^) produced more and relatively larger megadalton oligomer complexes than CHGA with elaborated O-glycans (HEK293^WT^) or without GAG chains (HEK293^ΔB4GALT7^). Lowering further to a pH of 4.5 pushed almost all CHGA from WT cells into the > 500 kDa megadalton complexes. At pH 4.5, a significant proportion of CHGA from HEK293^ΔB4GALT7^ and HEK293^ΔSC^ formed even larger complexes (>10.000 KDa) (Fig. 6*B*), whereas 30% to 50% of the molecules formed smaller <500 KDa complexes (Fig. 6*C*).

This apparent difference in oligomerization of CHGA prompted us to study DCG morphology and how truncation of glycosylation affected this. To this end, we performed ultrastructural analysis of the DCG in the glycoengineered SH-SY5Y cells using transmission electron microscopy and found that DCGs across the different glycoengineered cells appeared similar, with vesicles containing a dense core with a thin bright halo between the core and the membrane (Fig. 6*D*). However, quantifying DCG size revealed that the proportion of larger DCGs (>170 nm diameter) was significantly higher in cells with truncated O-glycans compared to WT cells (Fig. 6*E*). In cells with truncated GAG biosynthesis, a similar, but not statistically significant, proportion of larger granules was observed.

Another central role of the secretogranins in DCGs is to concentrate biogenic amines to mM levels (88). We therefore investigated the effects of loss of elaborated O-glycans and GAGs on the capacity for storage of neurotransmitters. Using a scintillation counter to quantify cellular uptake of tritiated noradrenaline, we found that, in SH-SY5Y cells, truncation of both types of glycans dramatically increased uptake (+66 ± 21% and +106 ± 21% compared to WT, respectively) of noradrenaline (Fig. 6*F*). We confirmed that the enhanced noradrenaline uptake was vesicular by the addition of the vesicular monoamine transporter VMAT-inhibitor reserpine, which reduced uptake to the same level in all cells.

Taken together, these results demonstrate that O-glycans modulate the ability of CHGA to assemble into multimers and regulate the DCGs’ capacity for neurotransmitters in the SH-SY5Y cells. Thus, establishing a novel role for O-glycans in regulating neurotransmitter biology.

## Discussion

Here, we set out to develop a deep O-glycoproteome to map the occurrence and positions of O-GalNAc glycans on neuronal proteins. These proteins are poorly represented in past O-glycoproteomics studies, and here, we demonstrate the power of analyzing tissues from multiple species, allowing us to combine datasets and greatly expand the neuronal O-glycoproteome. Our map outlines O-glycoproteins in the neuronal cell secretory pathway, and we highlight novel glycosites in proteins involved in complex neuronal processes, emphasizing the importance of studying specific tissue sources to capture relevant glycoproteoforms. Our study further identified DCG secretogranins as major glycoproteins and demonstrated that abrogated O-glycans or GAGs do not affect regulated protein secretion but increase the vesicular storage of noradrenaline and modulate oligomerization of granins (CHGA).

Studies of glycoproteomes (and other PTMs) pose unique challenges compared to proteomics, with the need to identify sites of glycan attachment and ultimately determine the structures of glycans attached at individual glycosites (20). Studies of GalNAc-type O-glycoproteomes are particularly challenging compared to N-glycoproteomics for several reasons: (1) a reliable predictive consensus sequence motif for O-glycosylation may not exist (*e.g.* like the NXS/T sequon for N-glycosylation) (35); (2) an enzyme capable of releasing all O-glycans leaving a protein scar (*e.g.* like PNGaseF for N-glycans and deamidation of Asn to Asp) (89) has not been identified; (3) universal enrichment of O-glycoproteins/peptides by lectins has not been identified (*e.g.* like the lectin mix used for N-glycopeptides (90)); and (4) O-glycosylation is differentially regulated by the large family of GALNTs necessitating in some cases analysis of glycosites on a cell- or tissue-specific basis (31). Several strategies have been introduced to enrich glycoproteins/peptides to circumvent these challenges, including capture by metabolic glycan tagging (91), periodate-oxidation of sialylated peptides (92), lectin chromatography (93) (with/without combination with genetic simplification of O-glycans (SimpleCells) (23)), and, more recently, use of O-glycoproteases (29, 94, 95). Each of these improvements has different limitations, yet they have all clearly advanced our insights into the O-glycoproteome and, to a lesser extent, structures of O-glycans at particular glycosites. Here, we took advantage of the knowledge that the, by far, most abundant O-glycan structures in neuronal cells are based on Core1 structures (T and (di)ST O-glycans) (54), which are readily enriched by PNA and Jacalin lectins (Fig. 1*A*). In addition, to cover protein sources as widely as possible, we included animal tissues and used sequence conservation in the human orthologous proteins combined with NetOGlyc4.0 predictions to expand the neuronal O-glycoproteome. Our expanded O-glycoproteome represents the most comprehensive to date (866 O-glycoproteins and 4228 O-glycosites), highlighting the wide occurrence of O-glycans on major classes of important neuronal proteins (Fig. 2).

A major motivation for this study was our recent findings that CDG caused by deficiencies in the *GALNT2* (33) and *GALNT11* (manuscript in prep) genes involves neurodevelopmental and intellectual impairments. In the case of GALNT11, the only non-redundant O-glycosylation functions known are the low-density lipoprotein receptor (LDLR) and related receptor (LRP) family that share O-glycosites in short linkers in between LDLR class A (LA) repeats in the ligand-binding domain, which are uniquely glycosylated by GALNT11 to modulate receptor affinities and specificities (16, 96). Gaining knowledge on O-glycosites in neuronal proteins is crucial to begin to understand how deficiencies in initiation of O-glycosylation underlie neuronal pathologies. Previous O-glycoproteomic studies of murine synaptosomes and human CSF contributed 118 and 60 glycoproteins, respectively (58, 97), and we later presented a strategy based on selective enrichment of low Mw glycoproteins, resulting in the identification of >90 glycosylated bioactive peptides (14). A more recent study of mouse brain tissue contributed around 100 O-glycoproteins and 200 O-glycosites (94). Our map greatly expands and increases the resolution of the neuronal O-glycoproteome, and collectively, these studies present a detailed view of the neuronal O-glycosylation landscape. One exciting finding is that the bulk of neuronal O-glycoproteins are known to play roles in different aspects of neuroplasticity, while only a minor fraction is associated with fast synaptic transmission. This contrasts the identified N-glycoproteins from similar tissues, where several N-glycosites are found on, *e.g.*, GABA and glutamate receptors (56, 57, 58). This surprising finding recapitulates recent findings by Cummings and colleagues (94), where the identified O-glycoproteins in mouse brain were also noted to be distinct from identified N-glycoproteins. Although these observations clearly need further investigation, it is noteworthy that N-glycoproteins involved in fast synaptic transmission are found with immature high-mannose structures (98) and therefore have been suggested to be secreted without passing the Golgi apparatus and thus not exposed to GALNTs and O-glycosylation in the Golgi.

To demonstrate the utility of our neuronal O-glycoproteome map, we pursued the finding of abundant O-glycosites in DCG-related proteins. We found that neuronal cell DCGs store and secrete numerous O-glycoproteins and identify the secretogranins as the major O-glycoprotein constituents. Secretogranins are highly abundant in the intragranular matrix (72, 99) and contribute to binding and concentration of neurotransmitters and Ca^2+^ to form high molecular weight aggregates as the granules mature and reduce in size (72, 100). This aggregation process occurs at acidic pH and is believed to drive the biogenesis of DCGs in the TGN (101). Glycosylation of proteins with granulogenic functions in other types of granules is known to play important structural roles in granulogenesis, such as O-glycosylation of PMEL in melanosomes (102), salivary glue protein three in salivary granules (12), and heparan sulfate on Serglycin in mast cell granules (103). For example, loss of the Serglycin proteoglycan in mast cells has dramatic consequences for granulogenesis and granular content, which is phenocopied by ablation of NDST1, important for HS sulfation (103). Inspired by this, we focused on CHGA, in which we identified up to 27 O-glycosites from a single biosource, expanding on previous studies that have reported up to 6 O-glycosites in this protein (29, 58, 94, 104, 105). The positions of these glycosites were distributed along the protein backbone and showed a high degree of variation between the investigated cell lines and tissues (Fig. 5). In contrast to the high number of identified glycosites, intact mass analysis of the recombinantly produced CHGA from HEK293 cells showed a collective occupancy of only ∼2 sites per molecule (Fig. S6) in agreement with a previous report that chromogranins from bovine adrenal medulla carry approximately 2.5 GalNAc glycans per molecule (5 μmol GalNAc per 100 mg Granins; bovine CHGA Mw: 48 mg/μmol) (106). Thus, the O-glycan profile of CHGA appears to be made up of several subsaturated O-glycosites in distinct positions that together add up to ∼2 glycans per molecule in a molecular average, rather than two fully occupied glycosites per molecule. This suggests a model where O-glycosylation sites serve to diversify the CHGA (and other secretogranin) gene products into a heterogeneous array of proteoforms that tailor the intragranular matrix to the specific neurotransmitters or hormones of the secreting cell.

In functional assays, we discovered that the pH-induced aggregation of CHGA into megadalton oligomers was diminished by loss of elaborated O-glycans as well as GAG chains (Fig. 6). Furthermore, DCGs from cells producing truncated O-glycans were significantly larger, consistent with a dysregulated granule maturation process. We detected multimerization at slightly lower pH than otherwise observed in mature secretory granules (pH 5.5) (107), likely explained by the dilute sample concentration (200 nM) employed in the mass photometry analysis, much lower than the concentration of CHGA in the TGN and mature DCGs (2–4 mM) (108). Finally, we found that truncation of both O-glycans and GAGs resulted in dramatic enhancement of the capacity to take up noradrenaline (Fig. 6). DCG size and neurotransmitter release have been demonstrated to correlate (109), and although we did not measure release in this study, we expect that the increased uptake relates to a dysregulated intragranular matrix that likely leads to the increased DCG size and predicts an increased quantal release from the glycosylation-deficient cells. Interestingly, CHGA and CHGB were recently demonstrated to undergo liquid-liquid phase separation (LLPS) in the early stages of granulogenesis with an impact on cargo recruitment. Von Blume and co-workers (110) showed that only the liquid state of CHGA and CHGB can recruit cargo, but not the aggregated state. Intriguingly, cytosolic O-GlcNAc glycosylation has been suggested to impact LLPS (111), and potential similar roles of O-glycosylation and GAGs in the dynamic LLPS of CHGA/B deserve further attention.

In summary, our targeted glycoproteomic analysis provides the most comprehensive map of neuronal O-glycoproteins to date, and we hope this will serve as a valuable resource for the community with a website-portal for convenient exploration (Neuronal Glycoprotein Family Viewer). The neuronal O-glycoproteome map provides a discovery resource for uncovering O-glycoprotein targets and molecular mechanisms underlying neuronal diseases, including brain cancer and neurodegenerative conditions as well as traits highlighted in GWAS, and neuronal phenotypes caused by CDG. The abundance of O-glycans on proteins involved in processes related to neuroplasticity underscores the potential of O-glycosylation in shaping the adaptability of neuronal circuits as well as their involvement in vesicular transport and secretion. Defining specific structure-function relationships remains a major barrier for understanding the full scope of functions of protein glycosylation. Here, we identified a new function of O-glycosylation in regulating DCGs and secretion of neurotransmitters, but the glycoproteome points to many other potential functions to be explored.

## Experimental procedures

### Ethical considerations

Experiments involving mice were conducted in accordance with the ethical guidelines of the United States National Institutes of Health and with the approval of the Institutional Animal Care and Use Committee of the University of Pennsylvania. CSF samples were collected with written informed consent, and subsequently pooled and anonymized. The National Committee on Health Research Ethics has evaluated that the use of these samples for the glycoproteomics study did not need approval because of the anonymization of patient samples. Studies in this work abide by the Declaration of Helsinki principles.

### Cell culture

SH-SY5Y cell lines (female) were cultured in 45% Dulbecco's modified Eagle's medium and 45% RPMI 1640 supplemented with 10% heat-inactivated fetal bovine serum (FBS) (Sigma–Aldrich) and 2 mM GlutaMAX (Gibco). STC-1 (gender unspecified, ATCC, CRL-3254), N2a (Male, ATCC, CCL-131), CAD (Male, ATCC, CRL-11179) and adherent HEK293 6E (Female, obtained through a license agreement with Dr Yves Durocher, Bioprocédés Institute de recherche en biotechnologie) cell lines were cultured in 90% Dulbecco's modified Eagle's medium (Sigma–Aldrich) supplemented with 10% heat-inactivated FBS (Sigma–Aldrich) and 2 mM GlutaMAX (Gibco). All cells were kept in a humidified incubator at 37 °C and 5% CO_2_. Suspension HEK293 6E cell lines were cultured in an orbital shaker in F17 medium (Invitrogen) supplemented with 0.1% Kolliphor P188 (Sigma–Aldrich) and 2% Glutamax (Gibco). All cell lines used in this study were routinely tested for *mycoplasma* contamination.

### O-GalNAc glycoproteome preparation

Murine synaptosomes were isolated from flash-frozen cortical tissue from C57BL/6J (Jackson Laboratory) background mice prepared by using modifications of a previously published protocol for Percoll gradient isolation (112) and synaptosome protein preparation for mass spectrometry analysis (113). Briefly, we homogenized ∼200 mg of flash frozen cortical tissue in 2 ml of isotonic sucrose solution (0.32 M Sucrose, 1 mM EDTA, 5 mM Tris-HCl, pH 7.4, 0.25 mM DTT) containing protease inhibitors (Roche) using a Dounce glass homogenizer, centrifuged at 1000g for 10 min at 4 °C to remove nuclei. The supernatant was diluted to 2 ml with isotonic sucrose solution and applied to a discontinuous Percoll gradient (3% vol/vol, 10% vol/vol, 15% vol/vol, and 23% vol/vol). The Percoll gradient was then centrifuged at 31,000g for 5 min at 4 °C. Fractions three and four were combined, diluted to 50 ml with isotonic sucrose solution, centrifuged at 20,000*g* for 30 min at 4 °C to remove Percoll. The resulting pellet was resuspended in acetone and the proteins precipitated *via* incubation in ice-cold acetone at −20 °C overnight. Proteins were isolated by centrifugation at 15,000*g* for 30 min at 4 °C, acetone removed, and pellet allowed to air dry. Protein pellets were stored at −80 °C until glycoproteomic analysis. CAD and SH-SY5Y total cell lysates were obtained by washing the monolayers of cells in ice-cold phosphate-buffered saline (PBS), scraping off the cells and adding 2 ml 0.05 to 0.1% Rapigest (Waters) to solubilize the cell pellet. The resulting suspension was sonicated and cleared by centrifugation.

The samples were subjected to the workflow described previously (14, 23, 27) using trypsin protein digestion and Jacalin agarose beads (binds galactosyl (β-1,3) N-acetylgalactosamine, T-antigen, or α-N-acetylgalactosamine, Tn-antigen (114)) for lectin weak affinity chromatography (LWAC). SH-SY5Y and mouse synaptosomes, 200 μg digest was labeled with TMT6plex (Thermo Scientific, 90061) according to manufacturer’s instructions prior to LWAC enrichment.

#### Mass spectrometry

The LC-MS analysis was conducted using an EASY-nLC 1000 UHPLC system (Thermo Scientific) connected to an Orbitrap Fusion mass spectrometer (Thermo Scientific) or an EASY-nLC 1200 UHPLC system (Thermo Scientific) connected to an Orbitrap Lumos mass spectrometer (Thermo Scientific) and operated in a positive mode as previously described (14). In brief, the nLC utilized a single analytical column set up, employing PicoFrit Emitters (New Objectives, 75 μm inner diameter) that were packed in-house with Reprosil-Pure-AQ C18 material (Dr Maisch, 1.9-μm particle size, 19–21 cm column length). A precursor MS1 scan (m/z 350–1700) of intact peptides was performed in the Orbitrap at a resolution of 120,000. This was followed by high-energy collision dissociation (HCD)-MS2 with the collision energy for HCD scans set to 27% ± 5% and electron transfer dissociation (ETciD)-MS2 fragmentations at a resolution of 60,000 for the five most abundant multiply charged precursors in the MS1 spectrum.

Pig, rat, and human glycoproteomes were processed as described in, where part of the identified sites (10% of all PSMs) were extracted and analyzed (deposited in MassIVE (MSV000085289) and PRIDE (PXD018560)).

All O-glycoproteomic data analyzed in this study is deposited in the PRIDE repository (PXD057996).

#### Data analysis of O-GalNAc glycoproteomes

MS data processing for all raw files was performed using Proteome Discoverer (PD) version 1.4 or 2.2 software (Thermo Fisher Scientific). Raw files were searched with Sequest HT search engine against concatenated human-specific (Uniprot, reviewed, canonical, 2013, 23.472 canonical entries) or mouse-specific (Uniprot, reviewed, canonical, 2014, 16.683 canonical entries) databases, allowing both full- and semi-specific trypsin cleavage. The precursor mass tolerance was set to 10 ppm and the fragment ion mass tolerance to 0.02 Da. Carbamidomethylation on cysteine residues, TMT6plex on peptide N-Terminus and Lysine were used as a fixed modification. Methionine oxidation as well as HexNAc and Hex(1)HexNAc(1) modification of serine and threonine residues was used as variable modification, with a maximum of 10 variable modifications per peptide. The minimum peptide length was set to five amino acids. Peptide FDR level was set to 1% and minimum number of unique peptides for protein identification was set to 1.

### Multiple sequence alignment

Human ortholog protein sequences (taxID: 9606) of animal glycoproteins (*Sus scrofa*, taxID 9823*; Mus musculus*, taxID 10090*;* and *Rattus norvegicus*, taxID 10116) were identified using the InParanoiDB algorithm (115). In cases where no homolog protein sequence was annotated in InParanoiDB, human homologs were identified by gene name in the human proteome and/or BLAST search of the protein sequence against the human proteome to determine homology.

Pairwise sequence alignments between animal and human orthologue sequences were performed using Clustal O (116) through the Biostrings R-package msa interface (117) (using default parameters and the Gonnet substitution matrix). Animal glycosites that aligned to a Ser, Thr or Tyr in the human sequence in the alignment were considered conserved and included in the expanded neuronal glycoproteome.

### Stimulated secretion

Five million cells per T75 flask were seeded in pairs of each genotype and cultured for 3 days. On the day of assay, cells were briefly washed in Krebs Ringer Buffer (KRB: 25 mM HEPES pH 7.4, 6 mM Glucose, 1.2 mM MgSO_4_, 2 mM CaCl_2_, 130 mM NaCl, 5 mM KCl, 0.2 μm sterile filtered) followed by four consecutive incubations in 10 ml KRB (4 × 30 min, 37 °C) to wash out endocytosed proteins from the FBS in the culture medium. Following this, both flasks in each pair were incubated (15 min at 37 °C) in 5 ml KRB and the supernatant collected as a reference secretome. Subsequently, to collect the depolarized and basal secretory products, one flask in each pair was incubated (15 min at 37 °C) in depolarizing conditions (5 ml KRB adjusted to 35 mM NaCl, 100 mM KCl) while the second flask was incubated in non-depolarizing conditions (KRB). The medium was collected and centrifuged (1.500*g* for 10 min at 4 °C) to remove cellular debris. The secretomes were adjusted to 0.2% NaDeoxycholate and proteins were concentrated by precipitation with ice-cold trichloroacetic acid (10% w/v, 2 h on ice, shaking) (118). The solution was spun at 10.000*g* 4 °C for 10 min, and deoxycholate was washed out of the pellet by two rinses with acetone pre-equilibrated to −20 °C. Secretome pellets were air-dried and kept at −80 °C until the day of assay.

### Multiplexing strategy for MS-based detection of regulated secretomes

To prepare secretome samples for mass spectrometric analysis, pellets as prepared above were resuspended in 120 μl 50 mM ammonium bicarbonate containing 0.1% Rapigest (Waters) by two rounds of heating (80 °C, 10 min) and sonication in a water bath (10 min). 20 μl aliquots were kept for Western blot analysis (see below). The proteins in the remaining 100 μl sample were reduced (5 mM DTT, 1 h, 60 °C), alkylated (10 mM iodoacetamide, 30 min, RT, in the dark), and digested by the addition of 250 ng trypsin (Roche, RTRYP-RO; 37 °C, overnight). The reaction was quenched by acidifying with trifluoroacetic acid (TFA). The resulting Rapigest precipitate was cleared by centrifugation (10,000*g*, 10 min) and peptides in the supernatant were purified on C18 Sep-Pak columns (Waters), followed by removal of solvent using a SpeedVac vacuum concentrator (Thermo Fischer Scientific). The dried peptides were resuspended in 60 μl MQ and peptide concentration measured on Nanodrop. 1 μg of each digested secretome was dried (SpeedVac vacuum concentrator) and kept at −80 °C until day of TMT labelling.

For TMT labeling, 1 μg of digested secretome was resuspended in 15 μl 200 mM HEPES pH 8. Peptides were labeled by addition of 80 μg TMT 10plex labeling reagent (Thermo Scientific, 90110) in 5 μl acetonitrile (1 h, RT, kept dark) followed by quenching in 0.25% hydroxylamine in a final volume of 25 μl (15 min, RT). The reaction was stopped by addition of 1% TFA and 0.9 μg of each TMT-10plex labeled digested secretomes were combined, dried by SpeedVac centrifugation, resuspended in 0.1% TFA and desalted on C18 StageTips (3M Empore). A total of 20 individual samples of WT and KO basal and depolarized secretomes necessitated multiple batches of 10plex analysis. To enable normalization and comparison between batches in the post-acquisition data analysis, an internal reference proteome (a WT polyclonal secretome) was included in each individual TMT-10plex assay. Each multiplex batch was subjected to high pH (Thermo Scientific, 84868) fractionation prior to nLC-MS/MS analysis.

LC-MS analysis was conducted as described for the glycoproteomes above, except HCD-MS2 fragmentations were acquired with the collision energy for HCD scans set to 37% ± 5% at a resolution of 60,000 for the 10 most abundant multiply charged precursors in the MS1 spectrum. A minimum MS1 signal threshold of 50,000 was set to trigger data-dependent fragmentation. A dynamic exclusion window of 30 s was used to avoid repeated analysis of the same species. Data analysis was performed as described above except that HexNAc and Hex(1)HexNAc(1) were not included as variable modifications, the Reporter Ion Quantifier node was applied for TMT quantification of reporter ions using total peptide level normalization and Trypsin digestion was restricted to full specificity with a maximum of two missed cleavages. All secretomics data analyzed in this study is deposited in the PRIDE repository: PXD057996.

#### Internal reference scaling

Quantitative analysis of secretomes was performed according to the workflow of Phillip Wilmart (https://github.com/pwilmart/IRS_normalization). In brief, protein abundances not represented in all channels or characterized only by at 1 PSM were removed, and the total protein abundance of the individual channels was normalized by scaling to the average total intensities across all 10 channels within a TMT 10plex assay (Sample loading normalization). To account for and quantify bias in reporter ion intensities between batches of TMT10plex assays, protein abundances within the pooled internal reference included in all TMT10plex assays were used to calculate scaling factors between batches on a protein-specific basis. This allows for the integration of all TMT-10plex assays in a single analysis pipeline. The internal reference scaling is described in detail in (82). Differential protein abundances between groups were analyzed using the Bioconductor package edgeR.

### LC-MS identification of GAG chain attachment sites

SHSY5Y^WT^ was seeded out in T175 flasks and grown to ∼ 80% confluency for 48 h. To rinse out serum proteins in the media, cells were washed 3× in PBS and were subsequently incubated in serum-free RPMI overnight to collect secreted proteins. On the following day, the media was collected and spun (5.000× G, 10 min, 4 °C), and the supernatant was frozen until day of assay. Culture media were reduced (5 mM DTT), alkylated (15 mM iodoacetamide) and subjected to trypsin digestion overnight (37 °C) with sequencing grade modified trypsin (Promega). The samples were then enriched for GAG-glycopeptides using SAX-chromatography (Vivapure, Q-mini H), desalted using C18 spin columns (8 mg resin, Pierce) and subjected to enzymatic depolymerization with 10 mU chondroitinase ABC (C3667, Sigma-Aldrich) and/or 5 mU heparinase II (no EC number), 5 mU heparinase III (EC 4.2.2.8) (both from *Pedobacter heparinus* overexpressed in *Escherichia coli*; kind gift from Prof. Jian Liu, University of North Carolina) at 37 °C for 16 h in digestion buffer (50 mM NH_4_OAc, pH 8.0. Reactions were supplemented with 4 mM CaCl_2_ when heparinase was used). The samples were again desalted, dried and stored at −20 °C until MS analysis.

The samples were analyzed on an Orbitrap Fusion Tribrid mass spectrometer coupled to an Easy-nLC 1200 system (Thermo Scientific), as previously described (87). Briefly, glycopeptides (3 μl i.v.) were trapped on an Acclaim PepMap C18 precolumn (20 × 0.1 mm I.D., 5 μm) and separated on an analytical column (350 × 0.075 mm I.D.) packed in-house with 3 μm Reprosil-Pur C18-AQ particles (Dr Maisch GmbH), coupled to a Flex nanospray ion source operating at 1.8 kV in positive mode. The gradient was run at 300 nl/min: 10 to 50% B-solvent (80% acetonitrile, 0.2% formic acid in dH_2_O) in A-solvent (0.2% formic acid in dH_2_O) over 60 min, 50 to 100% B over 5 min, with a final hold at 100% B for 10 min. MS scans were performed at 120,000 resolution (at *m/z* 200), an Automatic Gain Control (AGC) target value of 10^6^ with a mass range of *m/z* 600 to 2000. MS^2^ analysis was performed in a data-dependent mode with 3 s duty cycle time and dynamic exclusion for 10 s. The most abundant doubly or multiply charged precursor ions in each MS scan were fragmented by HCD at NCE levels of 20%, 30%, 35% and 40%. MS^2^ scans at each energy level were acquired separately: one microscan performed at 30,000 resolution (at *m/z* 200), AGC 5.0 × 10^4^ with a maximal injection time of 60 ms, a fixed first mass of *m/z* 100, an isolation window of 2.5 Da, maximum and minimum intensity threshold of 1.0 × 10^20^ and 1.0 × 10^4^, respectively. All GAG glycoproteomic data analyzed in this study is deposited in the PRIDE repository: PXD058040.

#### Data processing of O-Xyl attachment sites

The MS data were processed using Mascot Distiller and database searches for GAG-glycopeptides were performed as described (119). Briefly, Mascot distiller (version 2.6.1.0, Matrix Science) was used for conversion of HCD.raw spectra into singly protonated peak lists in Mascot.mgf format. Database searches were performed using an in-house Mascot server (version 2.5.1) with enzyme specificity set to Trypsin, and to Semitrypsin, allowing for up to two missed cleavages, in subsequent searches of the Swissprot database (June 2019). The following constraints were included: peptide tolerance, 10 ppm; fragment tolerance, 20 ppm; fixed carbamidomethyl modifications of Cys residues and variable Met oxidation. For glycopeptide identifications, variable modifications of Ser residues with the residual hexasaccharide structure [HexA(–H_2_O)HexNAcHexAHexHexXyl-*O*-] without (C_37_H_55_NO_30_, 993.2809 u), with one (C_37_H_55_NO_33_S, 1073.2377 u), or with two (C_37_H_55_NO_36_S_2_, 1153.1945 u) sulfate groups attached. All Mascot-generated GAG-glycopeptide hits were manually evaluated according to previously established criteria (119).

### Immunocytochemistry

SH-SY5Y and STC-1 cells were seeded on sterile coverslips coated with type I collagen (1 μg/ml, O/N) and cultivated for 2 to 3 days. Cells on coverslips were washed (2× PBS) followed by fixation in 4% PFA (15 min, RT). The PFA was quenched by two washes in PBS^++^ (PBS supplemented with 1 mM CaCl2, 0.5 mM MgCl2) containing 20 mM Glycine and 50 mM NH_4_Cl followed by two washes in regular PBS^++^. Cells were permeabilized and blocked simultaneously in PBS++ containing 0.1% BSA and 0.2% Saponin (20 min, RT), and desialylated using 100mU/ml Neuraminidase (Sigma-Aldrich, N3001) in PBS^++^ containing 0.1% BSA (45 min, 37 °C). Cells were subsequently incubated with VVA- or PNA-biotin (Vector Labs, B-1235–2/B-1075–5,1:10.000) for 1h at RT. Primary probes were washed out (3× PBS^++^), and cells were incubated with secondary streptavidin-Alexa fluor 488 (Invitrogen, S32354, 1:500) incubated 1h at RT, kept dark. Finally, coverslips were washed (2× PBS and 2× MQ) and mounted in Prolong Gold anti-fade (Thermo Scientific, P36930). Images were acquired on an epifluorescence microscopy system (Nikon ECLIPSE Ts2).

### SDS-PAGE and immunoblotting

Samples resuspended in 1× LDS, 1× Red agent (NuPAGE) were boiled (10 min, 80 °C) and separated on 4% to 12% Bis-tris gels (NuPAGE, Thermo Scientific, NP0349BOX). Proteins were either visualized directly with InstantBlue (Expedeon, ISB1L) or blotted onto nitrocellulose membranes (1h at 320 mA). Membranes were blocked for 1h in TBS-T containing 1% milk, 0.1% BSA for protein blots or 2.5% BSA for lectin blots. In case of lectin binding, an additional desialylation step was performed where blots were incubated in PBS supplemented with 100 mU/ml neuraminidase (Sigma-Aldrich, n3001) for 1h at 37 °C. Subsequently, blots were incubated O/N using the following lectins or antibodies: VVA-Biotin 1:2000 (Vector-labs, B-1235–2), PNA-Biotin 1:2000 (Vector-labs, B-1075–5), Anti-CHGA 1:1000 (AbCam, Ab15160), Anti-CS 1:1000 (Abcam, ab11570), Anti-B-actin 1:10.000 (Protein Tech, 66009-1-Ig), anti-CHGB (Abcam, ab12242) or anti-VGF (Abcam, ab69989). Next, blots were washed in TBS-T (3 × 5 min) followed by 1 h incubation with secondary HRP-conjugated goat anti-rabbit Igs (DAKO, P0448, 0.25 μg/ml) or HRP-conjugated streptavidin 1:4000 (Invitrogen, S911). The membranes were washed in TBS-T (3 × 5min) and developed using SuperSignal West Pico PLUS Chemiluminescent Substrate (Thermo Scientific, A43841). Gels and blots were visualized using a LAS-3000 instrument (GE Healthcare). The specificity of CHGA, CHGB, and VGF recognizing antibodies was verified by ectopic overexpression in HEK293 6E cells (these cells do not express these proteins endogenously). The specificity of the anti-CS antibody was characterized previously in Chen *et al.*, 2018 (84).

Secretome pellets analyzed by electrophoresis were obtained as described above and directly resuspended in 1× LDS, 1× Red agent (NuPAGE, NP0007/B0009). To control for protein load, an aliquot was subjected to SDS-PAGE and relative protein concentration was calculated based on measurements of band intensity in each lane using ImageJ software (NIH). The remaining samples were adjusted to the same relative concentration and equal amounts of protein were subjected to both SDS-PAGE and Coomassie staining as well as immunoblotting.

For analysis of cell lysates, cells cultured in 6 wells were washed (3× PBS, 37 °C) and harvested in 0.5 ml RIPA buffer (150 mM sodium chloride, 1.0% Triton X-100, 0.5% sodium deoxycholate, 0.1% SDS (sodium dodecyl sulfate), 50 mM Tris, pH 8.0). The solution was cleared from cellular debris by centrifugation (21,000*g*, 10 min, 4 °C), and supernatant protein concentration was determined with Pierce BCA Protein Assay Kit (Thermo Scientific, 23225) following the manufacturer’s instructions. Samples were normalized to the same concentration prior to immunoblotting as described above.

Secreted proteins from the cell lines in question were obtained by culturing cells in reduced serum media (OptiMem, Thermo Scientific) for 48 h after which the medium was harvested. 17 μl conditioned media was adjusted to 20 mM Tris pH 6.8 and treated with or without 5U of SialExo and/or OpeRATOR (Genovis, G2-OP1-020) for mucinase assays, and 0.25mU/μl chondroitinase ABC (Sigma, C3667) for chondroitinase assays in a final volume of 20 μl (37 °C, O/N). The reaction was stopped by adjusting the reaction to 1× LDS, 1× Red agent (NuPAGE, NP0007/B0009) and subjected to immunoblotting as described above. For recombinant proteins, 2 μg of purified product was used in place of media and subjected to SDS-PAGE and Coomassie staining as described above. Quantification of band intensity was performed using ImageJ (NIH). The CS-CHGA band was expressed as the fraction of the total signal within the same lane. O-glycosylated CHGA was expressed as the total signal within the OpeRATOR and neuraminidase-treated lane as a fraction of the total signal of the non-treated lane.

### Generation of isogenic CRISPR-knockout neuronal cell lines

CRISPR/Cas9 KO was performed using the GlycoCRISPR resource containing validated gRNA libraries for targeting of human glycosyltransferases (120). In brief, SH-SY5Y and STC-1 cells (2 x 6 wells) were grown to ∼70% confluency and co-transfected with plasmids containing gRNA and GFP-tagged Cas9-PBKS (1 μg of each plasmid/transfection) using either lipofectamine 3000 transfection agent (ThermoFisher Scientific, L3000015) for STC-1 according to manufacturer’s protocol or electroporation with AMAXA (Lonza, KitV, VCA-1003, Program A23, two million cells) for SH-SY5Y cells. Forty-eight hours post-transfection, cells were bulk-sorted based on GFP expression by FACS (SONY SH800). At 70% confluency, bulk cultures were single-cell-sorted (STC-1) or sorted to five cells per well (SH-SY5Y) into 96-well plates. Cell clones were screened for knock out of the target gene by Indel Detection by Amplicon Analysis (IDAA) (121) with primers amplifying gRNA targeting sites. Final clones were further validated by Sanger sequencing of the amplified region.

### Validation of COSMC2

A plasmid containing the *COSMC2* sequence (Human, Uniprot: P0DN25) with a C-terminal “GGG”-linker followed by a FLAG tag was obtained from Genewiz (www.genewiz.com) in the pcDNA3.1Zeo^+^ framework. N2a cells were cultured to 70% confluency and stably transfected using Lipofectamine 3000 according to manufacturer’s protocol and subsequently maintained in 700 μg/ml Zeocin (Invitrogen, Sigma). Cells were seeded on coverslips and grown for 2 days before staining with lectins as described above.

For flow cytometry analysis, cells were first incubated with 100 mU/ml neuraminidase (Sigma-Aldrich, n3001) for 1 h at 37c. Following 3× wash steps, cells were stained with biotinylated PNA (0.2 μg/ml) or VVA (0.2 μg/ml) lectins (Vector Laboratories) on ice or at 4  °C for 1 h. Subsequently, cells were washed 3× and stained with Alexa Fluor 647-conjugated streptavidin (1:1000) (Invitrogen, A-21245) 4  °C for 1 h. After a final set of three washes, cells were resuspended in PBA for flow cytometry analysis (SONY SA3800). All steps were carried out in PBS containing 1% BSA (w/v). Mean fluorescent intensity (MFI) was quantified using the SONY SA3800 software.

### Recombinant production and purification of CHGA glycoforms

CHGA (Uniprot P10645) cDNA cloned into pCMV6-Entry was purchased from Origene (#RC200492). We modified this plasmid by annealed oligo cloning to introduce a C-terminal TEV protease site followed by a His_6_-tag and stop codon. The resulting CHGA-TEV-His_6_ open reading frame was subsequently In-Fusion (Takara) cloned into *pIRES-Puro3* (Takara, 631619), generating *pIRESpuro-CHGA-TEV-His*_*6*._ Plasmid sequences were verified by Sanger sequencing. To establish cells with stable expression of CHGA-TEV-His_6,_ HEK293 6E^WT^ cells were cultured in 24-well plates for 24 h and transfected with 250 ng of pIRESpuro-CHGA-TEV-His6 using Lipofectamine 3000 according to the manufacturer's instructions. The following 10 days, cells were selected for expression of CHGA-TEV-HIS_6_ by culturing in medium supplemented with 1 μg/ml of puromycin (Invivogen, ant-pr-1). After 10 days, the puromycin concentration was reduced to 0.25 μg/ml. Stable CHGA-TEV-HIS_6_ expressing clones were obtained by single-cell sorted by FACS (Sony SH800). Clones with the highest protein expression were screened by direct enzyme-linked immunosorbent assay (ELISA) using anti-6xhis-HRP (Invitrogen, R931-25).

Select glycosyltransferases were targeted in HEK293 6E^WT^-TEV-His_6_ by CRISPR knockout as described above, except transfection was performed in 24-wells by addition of 100 μl containing 3 μg PEI (Polysciences, 23966-1) and 0.5 μg of each plasmid containing gRNA and GFP-tagged Cas9-PBKS.

For production and purification of CHGA wild type and glycoforms, HEK293 6E cell lines stably expressing CHGA-TEV-His_6_ were seeded at a density of 0.5 × 10^6^ cells/ml and cultured for 4 days on an orbital shaker in F17 medium (Gibco) supplemented with 0.1 Kolliphor P188 (Sigma-Aldrich) and 2% Glutamax (Gibco). Conditioned media containing secreted CHGA-TEV-His_6_ was harvested (500*g*, 10 min followed by 4000*g*, 10 min), adjusted to 25 mM Tris pH 7.5, 300 mM NaCl and 10 mM Imidazole, and frozen until the day of purification. Nickel affinity purification was performed by addition of 1 ml nickel-nitrilotriacetic acid (Ni-NTA) beads (Qiagen, 30,210)/30 ml culture medium and incubation rolling over night at 4 °C. The following day, the beads were captured on a column, washed with 2 × 10 column volumes of washing buffer (25 mM tris, pH 7.5, 300 mM NaCl) and CHGA-TEV-His_6_ was eluted in washing buffer supplemented with 100 mM imidazole. Eluted fractions were analyzed by SDS-PAGE, and fractions containing the CHGA-TEV-His_6_ were desalted followed by buffer exchange to 50 mM NaCl, 10 mM HEPES pH 7.4 using 10 kDa molecular weight cutoff columns (Vivaspin, VS0602). Yields were quantified using a Pierce BCA Protein Assay Kit (Thermo Scientific, 23225) following the manufacturer’s instructions. HIS_6_-tag was removed by TEV-protease digestion (5U enzyme pr 100 μg protein, Sigma, T4455), overnight at 30 °C, 50 mM NaCl, 10 mM HEPES pH 7.4. The removal of the HIS_6_-tag was confirmed by immunoblot against CHGA and C-terminal HIS6 (Invitrogen, R931-25) as described above. Free HIS_6_-tag was removed by reversed phase HPLC on a C4 column (Jupiter, 5 cm; ID:4.6; 300Ă, Phenomenex) using a 0 to 100% gradient of 90% acetonitrile in 0.1% TFA, yielding pure CHGA. Subsequently, organic solvent was removed using a SpeedVac vacuum concentrator (Thermo Scientific).

Molecular weight of purified Chromogranin A was evaluated using MALDI-TOF-MS in linear positive mode on a Bruker Autoflex instrument (Bruker Daltonik GmbH, Bremen, Germany) calibrated using Protein Standard ii (Bruker Daltonik GmbH, Bremen, 8207234, Germany) by mixing 1 μg of protein with a saturated solution of α-Cyano-4-hydroxycinnamic acid in acetonitrile/H_2_O/TFA (70:30:0.1) at a 1:1 ratio on a target steel plate.

### Mass photometry

Chromogranin A complex formation at different pH values was measured on a mass photometry instrument (OneMP, Refeyn Ltd, Oxford, UK). Landing events were recorded at 200 nM working protein concentration at various pH values using the following buffers: 20 mM HEPES (pH 7.5, 7.0); 20 mM MES (pH 6.5, 6.0); and 20 mM NaAcO (pH 5.5, 5.0, 4.5), each supplemented with 50 mM NaCl. Measurements were performed using the following procedure: Prior to protein loading, the focus plane was found in 10 μl buffer alone, after which 10 μl of 2× concentrated protein solution (400 nM) in the same buffer was mixed by resuspending 3 times. Recordings of landing events of one- or 2-min length were acquired in Acquire MP immediately after protein loading.

Before each session, the machine was calibrated using Native Mark Unstained Protein Standard (MWs 66 kDa, 146 kDa, 242 kDa, and 480 kDa, ThermoFisher, LC0725) in 20 mM HEPES, 50 mM NaCl, pH 7.5 following the same procedure described above. For all experiments, measurements of protein solutions were performed in clean wells of a reusable gasket (Reusable Culturewell gasket 3 mm diameter × 1 mm depth, Sigma, GBL103250) mounted on Glass slides (GLW storing systems, ZK25) that were installed in the machine using immersion oil (Thorlabs, MOIL-30). The glass slides and gaskets were cleaned with alternating rinses of water and isopropanol eight times each and dried with compressed air prior to each experiment.

Analysis of each movie was performed with the mass photometry software, DiscoverMP. The contrast measurement of each calibration standard was identified and presented as histograms with fitted gaussian curves used to determine the complex mass of chromogranin A based on the contrast of each landing event. Number of molecules were calculated by dividing mass with the mass of the primary sequence of chromogranin A without his-tag (50.438 kDa).

### Transmission electron microscopy

0.5 million SH-SY5Y cells were grown for 48 h on Thermanox coverslips (Thermo Scientific, 174,950). Cells were washed 3× in PBS and fixed for 10 min in 4% PFA in PBS followed by postfixation with 2% glutaraldehyde in 0.05 M sodium phosphate buffer (pH 7.2). The samples were rinsed three times in 0.15 M sodium phosphate buffer (pH 7.2) and subsequently postfixed in 1% w/v OsO4 with 0.05 M K_3_Fe(CN)_6_ in 0.12 M sodium phosphate buffer (pH 7.2) for 2 h. The specimens were dehydrated in graded series of ethanol, transferred to propylene oxide and embedded in Epon according to standard procedures. Sections, approximately 60 nm thick, were cut with a Ultracut 7 (Leica) and collected on one hole copper grids with Formvar supporting membranes, stained with uranyl acetate and lead citrate, and subsequently examined with a Philips CM 100 Transmission EM (Philips,), operated at an accelerating voltage of 80 kV. Digital images were recorded with an OSIS Veleta digital slow scan 2k x 2k CCD camera and the ITEM software package. Images were acquired blinded at 24.500× magnification. DCG size was measured using ImageJ (NIH) software before unblinding and analysis of granule size distributions.

### Measurement of cellular uptake of tritiated noradrenaline

Two million SH-SY5Y cells were seeded in 6-well plates and incubated for 2 days. On the day of assay, the cell culture media was replaced by 1 ml buffer (5 mM KCl, 135 mM NaCl, 0.2 mM Ascorbic acid, 0.2 mM pargyline [Sigma]) containing 50 nM [3H]-Noradrenaline (12.4ci/mmol, PerkinElmer, NET377250UC) and incubated for 1 h at 37 °C. To control for vesicular uptake, replicate wells were co-incubated with 10 μM reserpine (Sigma). Subsequently, excess isotope was rinsed out by 6× washes in the same buffer supplemented with 1 μM desipramine (Sigma). Cellular [3H]-Noradrenaline was recovered by a brief incubation of the cells in 1 ml extraction buffer (0.4 M Perchloric acid). The extracted solution was mixed with 3.5 ml Ultima Gold (PerkinElmer, 6013326), and scintillation was measured on Tri-Carb 4910 TR (PerkinElmer) for 5 min.

To correlate uptake to protein concentration, replicate plates were set up in parallel and treated the same way, except no [3H]-Noradrenaline was added in the uptake buffer. Proteins were extracted in 1 ml RIPA buffer (150 mM sodium chloride, 1.0% Triton X-100, 0.5% sodium deoxycholate, 0.1% SDS, 50 mM Tris, pH 8.0), and the resulting solution was cleared from cellular debris by centrifugation (10 min, 21,000*g*). Protein content was measured using the Pierce BCA protein assay kit (Thermo Scientific, 23225).

### GO-term analysis

GO-term analysis was performed using agotool (122) by comparing all identified human glycoproteins (the corresponding human homolog was used for non-human proteins) to the entire genome. GO-term clustering was performed using the r-studio package “rrvgo” (123) on enriched cellular component GO-terms (FDR >0.05). The threshold for parent term generation was set to 0.95.

### Statistical analysis

All parameters and errors were determined with the R Studio software (Version 2022.07.0 Build 548, R version 4.2.1).

Significance in comparisons between secretomes was assessed using Fishers exact test. Benjamini Hochberg correction of *p*-values was employed for multiple testing for non-depolarized vs polarized secretomes and Bonferroni correction of alpha (*p*-value threshold) was employed for multiple testing for WT vs KO stimulated secretomes.

When comparing multiple groups, one-way ANOVA was performed with Tukey’s *post hoc* test for testing difference between multiple groups (ns = *p* > 0.05; ∗∗∗*p* < 0.001; ∗∗*p* < 0.01; ∗*p* < 0.05. Comparison of exactly two groups was performed with two-tailed Students *t* test (ns = *p* > 0.05; ∗∗∗*p* < 0.001; ∗∗*p* < 0.01; ∗*p* < 0.05).

### Additional resources

Web-based platform for exploration of the presented neuronal O-glycoproteome: https://neuronal.glycomics.ku.dk/.

## Data availability

Further information and requests for resources and reagents should be directed to and will be fulfilled by the lead contact, Katrine Schjoldager (Schjoldager@sund.ku.dk).

All the cell lines generated in this manuscript will be made available upon request. A material transfer agreement will be required prior to sharing of materials. All plasmids, gRNAs and primers used in this study are listed in Table S10.

Proteomics and glycoproteomics data have been deposited in the PRIDE database [O-glycoproteomes and secretomes: PXD057996, GAG-glycoproteomes: PXD058040] and are publicly available as of the date of publication. Pride accession numbers are also listed in the key resources table.

TMT-analysis was done according to the internal reference scaling method for which the code is available at https://github.com/pwilmart/IRS_normalization.

Any additional information required to reanalyze the data reported in this paper is available from the lead contact upon request.

## Supporting information

This article contains supporting information (32, 53, 120).

## Declaration of generative AI and AI-assisted technologies

No AI or AI-assisted technologies were used in this work.

## Conflict of interest

The authors declare that they have no conflicts of interest with the contents of this article.
