# Supplementary material for: Map of the neuronal O-glycoproteome reveals driver functions in the regulated secretory pathway[image]
Source: J Biol Chem. 2025 May 29;301(7):110313. doi: 10.1016/j.jbc.2025.110313 (PMC12269846; doi:10.1016/j.jbc.2025.110313)
Supplement: Supporting figures [file mmc2.docx]

**Supporting Figure 1**

**
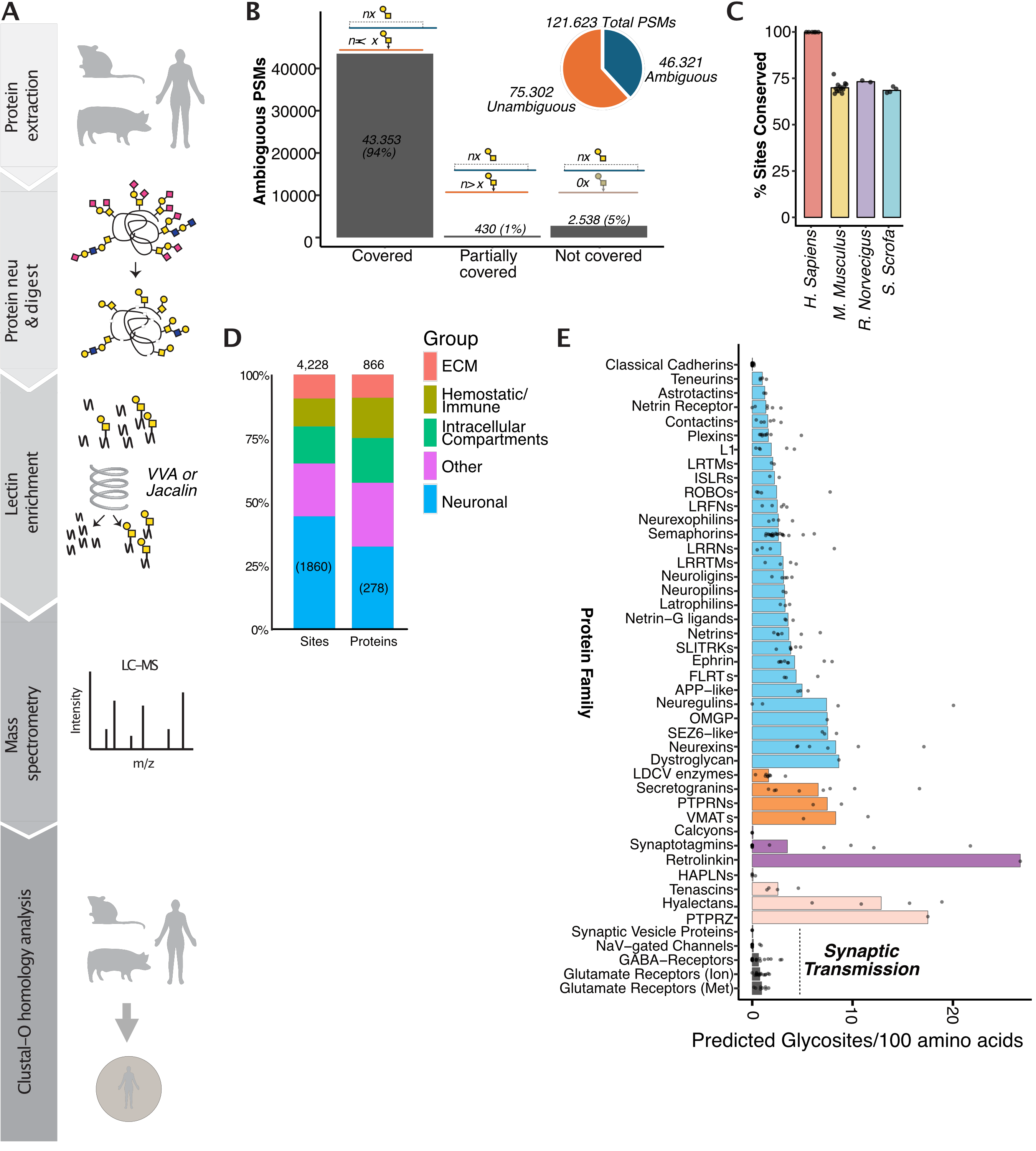
**

**Supporting figure 1. Glycoproteomics extended.**

(**A**) Overview of glycoproteomics workflow. Neu: neuraminidase (**B**) Number of PSMs containing one or more ambiguously assigned glycosites that are: a) all covered, b) partly covered, or c) not covered at all in the total pool of unambiguous PSMs. Pie diagram shows the fraction of PSMs that carry ambiguous/unambiguous site information. (**C**) Bar graph showing the mean percentage of sites that align to a S/T/Y in the human sequence between each glycoproteomic assay within the different species analyzed. Each datapoint represents a single glycoproteomic assay and demonstrates that the proportion of conserved and non-conserved sites was similar between datasets and species. **(D)** Distribution of the 4,228 sites in the expanded dataset belonging to 866 human consensus proteins within major protein groups. (**E**) Bar graph showing the average number of predicted glycosites (Net-O-Glyc 4.0) per 100 extracellular amino acids within each family. Each dot represents a single protein.

**Supporting Figure 2**


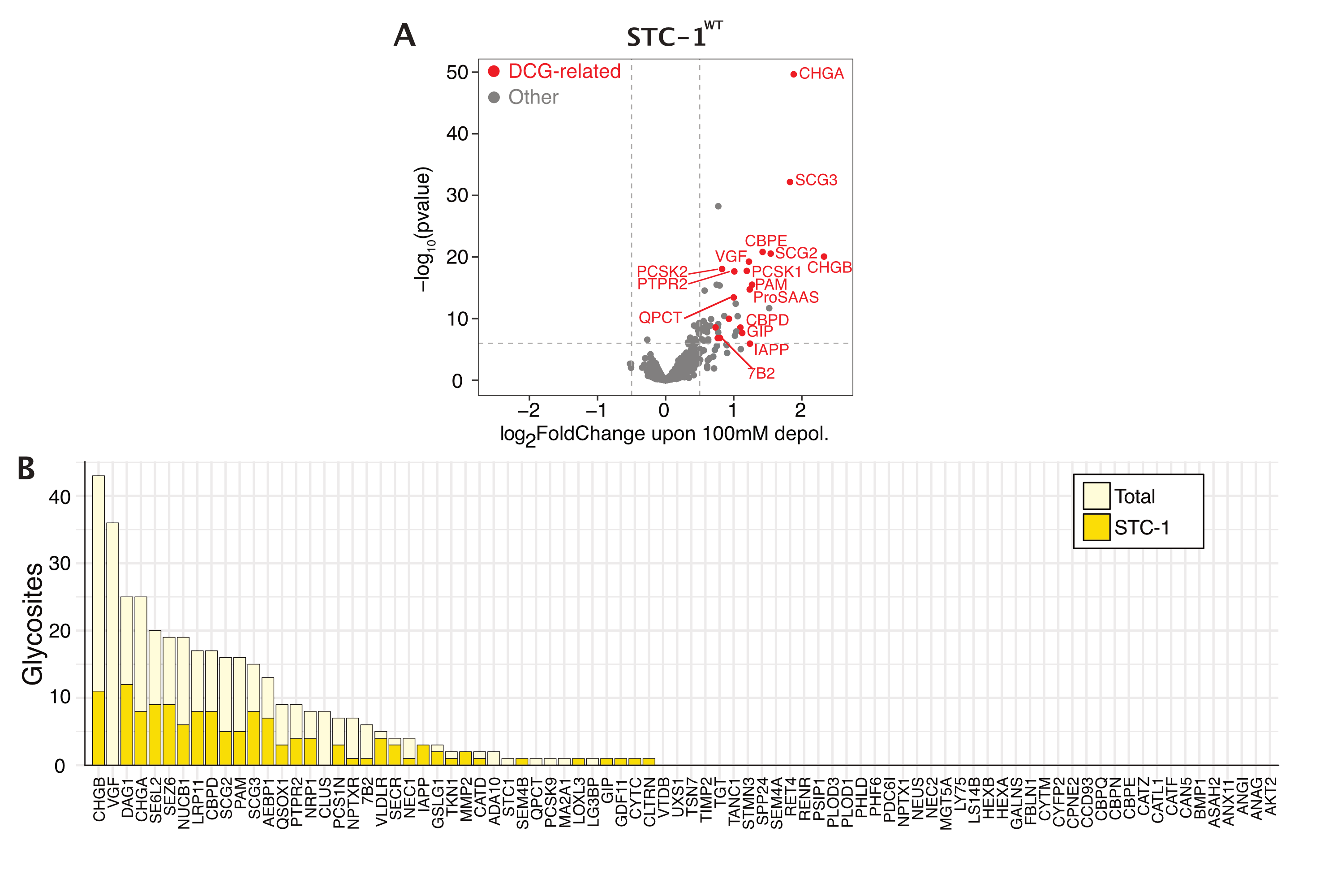


**Supporting figure 2. O-glycosylation within the regulated secretome**(**A**) Volcano plot showing log_2_ fold change of regulated secreted proteins after depolarization of STC-1 cells (n=5) with significantly (p<0.1) DCG-related regulated secreted proteins highlighted in red. (**B**) Stacked bar plot showing total numbers of O-glycosites found on regulated secreted proteins, distinguishing O-glycosites from the expanded dataset (Total) and experimentally identified expanding sites in STC-1 cells (STC-1).

**Supporting Figure 3**

**
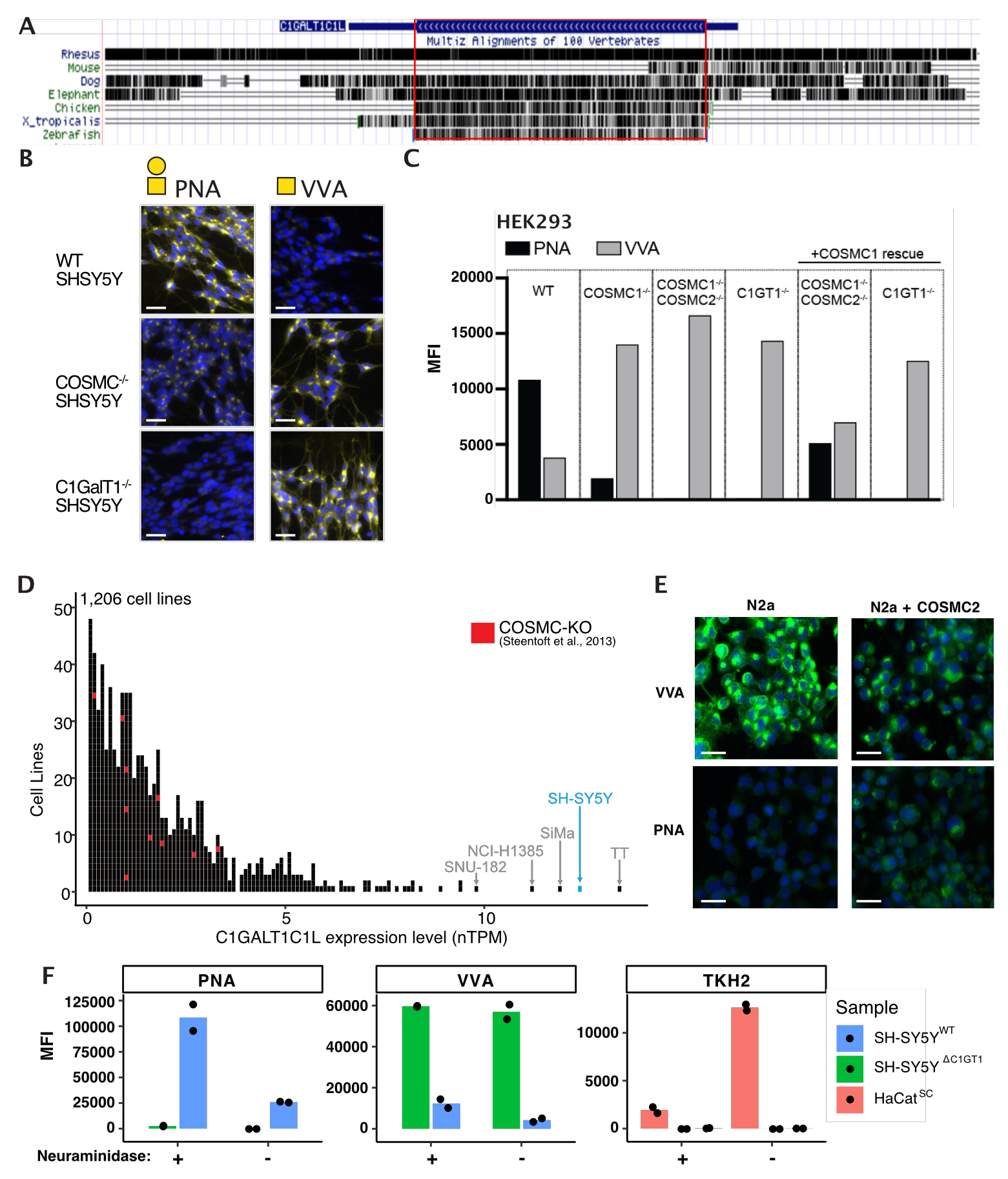
**

**Supporting figure 3. COSMC2 partially rescues the chaperone function of COSMC for C1GALT1
(A)** Excerpt from UCSC genome browser (<https://genome.ucsc.edu/>) showing the genomic conservation of COSMC2 (*C1GALT1C1L*) between species. The red box highlights the single COSMC2 exon and shows that the gene is deleted in the mouse. **(B)** Lectin staining of desialylated (+ neu) SHSY-5Y^WT^, SHSY-5Y^ΔCOMSC^ and SHSY-5Y^ΔC1GALT1^ cell lines probed with VVA or PNA that recognize the truncated Tn or elongated Core 1 structures, respectively, shows that the residual Core 1 activity could be abolished by targeting C1GALT1 directly. While we successfully generated a single isolated SH-SY5Y^ΔC1GALT1^ clone, we opted to target COSMC directly in SHSY5Y, where we successfully generated 5 individual cell clones to account for biological variation. **(C)** Cell surface PNA and VVA lectin stain of desialylated (+ neu) HEK293 cell lines quantified by flow cytometry. Note that the residual PNA signal in the ΔCOSMC1 was removed completely by Δthe COSMC1/2 double KO. **(D)** Excerpt from Human Protein Atlas (Sjostedt et al., 2020) displaying expression level of *COMSC2* in commonly used human cell lines including cell lines where targeting the chaperone *COSMC* has successfully resulted in truncation of O-glycan biosynthesis (red bars (Steentoft et al., 2013)). The red arrow indicates that the SH-SY5Y cell line has the second highest expression of *COSMC2* of all tested cell lines, explaining why this residual activity has not been observed previously. **(E)** Lectin staining, as in **B,** of the murine N2a cell line that is naturally COSMC-deficient, before and after recombinant expression of COSMC2. Note that PNA-staining was partially rescued, suggesting that COSMC2 can chaperone C1GALT1 in mouse cells as well. **(F)** Flow cytometry data as in **C** for SH-SY5Y^WT^ and SH-SY5Y^ΔC1GT1^ cell lines probed with VVA, PNA and TKH2 (recognizing STn), confirming lack of STn in the SH-SY5Y cell line. HaCat^SC^ was used as positive control for the TKH2 staining. Scale bars represent 20µm.

**Supporting Figure 4**


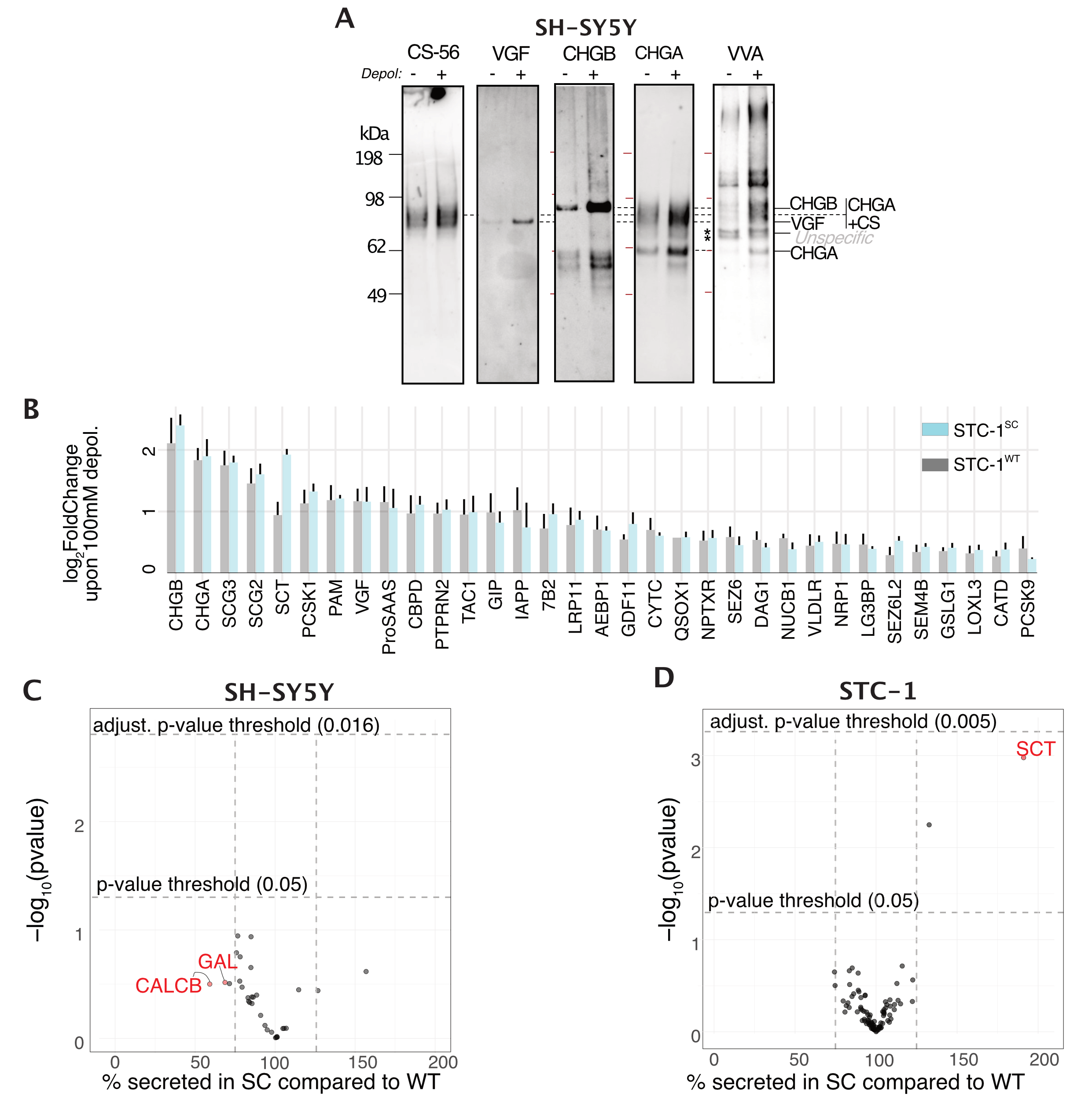


**Supporting figure 4. O-glycosylation within the regulated secretome**  (**A**)Western blot of conditioned medium collected from SH-SY5Y^SC^ treated for 15 min with either non-depolarizing (5 mM KCl) or depolarizing (100mM KCl) conditions. The secretome was probed for DCG-related proteins carrying the highest amount of O-glycosites (VGF, CHGA and CHGB) as well as for chondroitin sulfate (CS-56) and VVA for proteins carrying truncated O-glycans. (*) denotes background bands. (**B**) Bar plot showing the mean relative secretion of the KCl-responsive proteins in STC-1^WT^ and STC-1^SC^. Error bars represent + standard error of the mean, n = 5 clones. (**C-D**) Volcano plot showing percent change of stimulated secretory proteins in depolarized medium of SC over WT cell lines of SH-SY5Y and STC-1 with significance threshold annotated (p>0.05) as well as the Bonferroni adjusted significance threshold.

**Supporting Figure 5**


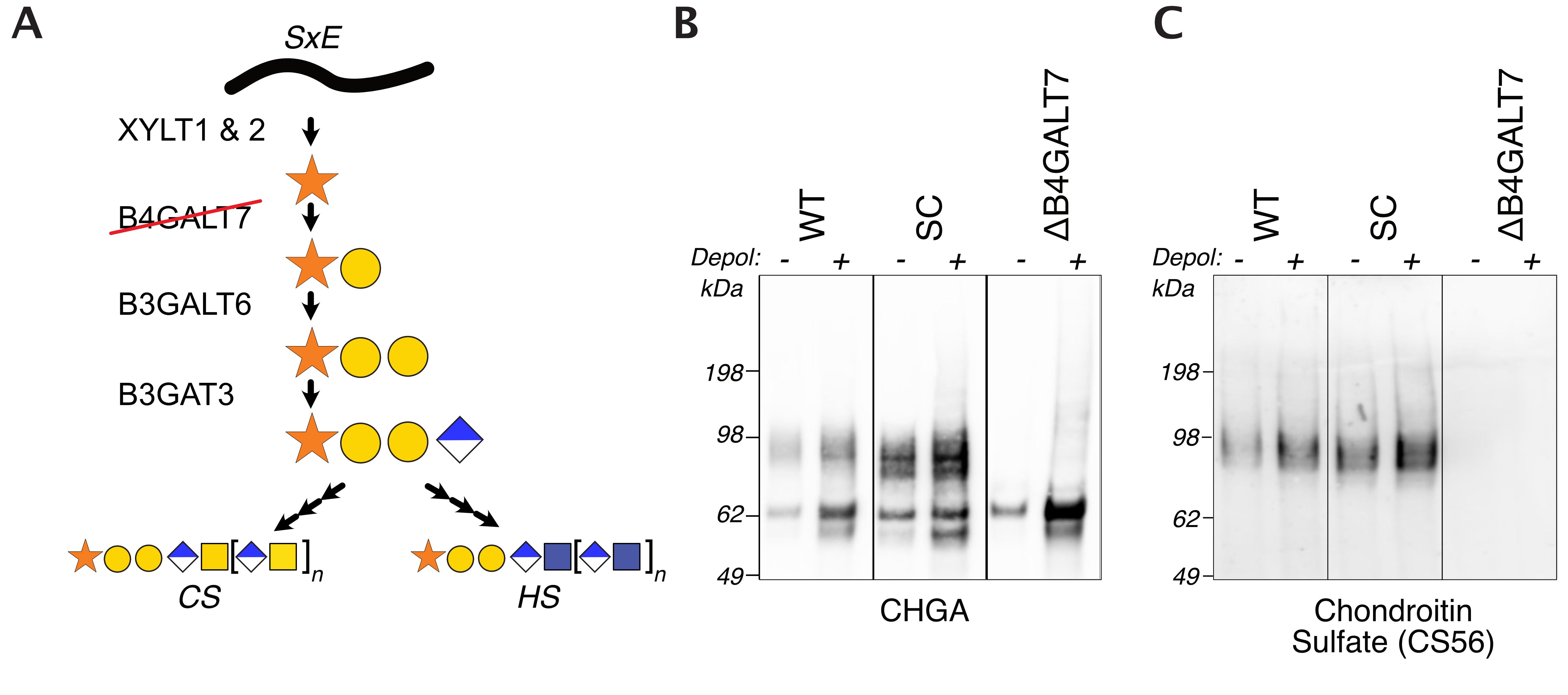


**Supporting figure 5. Investigating GAGs in the SH-SY5Y cell lines.**

(**A**) Schematic diagram of the GAG biosynthetic pathway and genetic engineering strategy to produce SH-SY5Y^ΔB4GALT7^ cell lines. GAG biosynthesis of the common tetrasaccharide linker (GlcA-β1-3Gal-β1-3Gal-β1-4Xyl-β1-*O*-Ser) is initiated by XYLT1 and XYLT2 and elongated by B4GALT7, B3GALT6 and B3GAT3. KO of B4GALT7 by CRISPR-Cas9 gene targeting resulted in cell lines only producing O-Xyl truncated immature GAGs.

(**B & C**) Western blot of depolarized (100mM KCl) or non-depolarized (5mM KCl) secretomes of SH-SY5Y^WT^, SH-SY5Y^SC^ or SH-SY5Y^ΔB4GALT7^ cell lines probed with anti-CHGA (**B**) and mAb CS56 to chondroitin sulfate (**C**) showing that the one major chondroitin sulfate smear co-migrates with the CHGA-GAG smear that is lost after B4GALT7 KO.

**Supporting Figure 6**


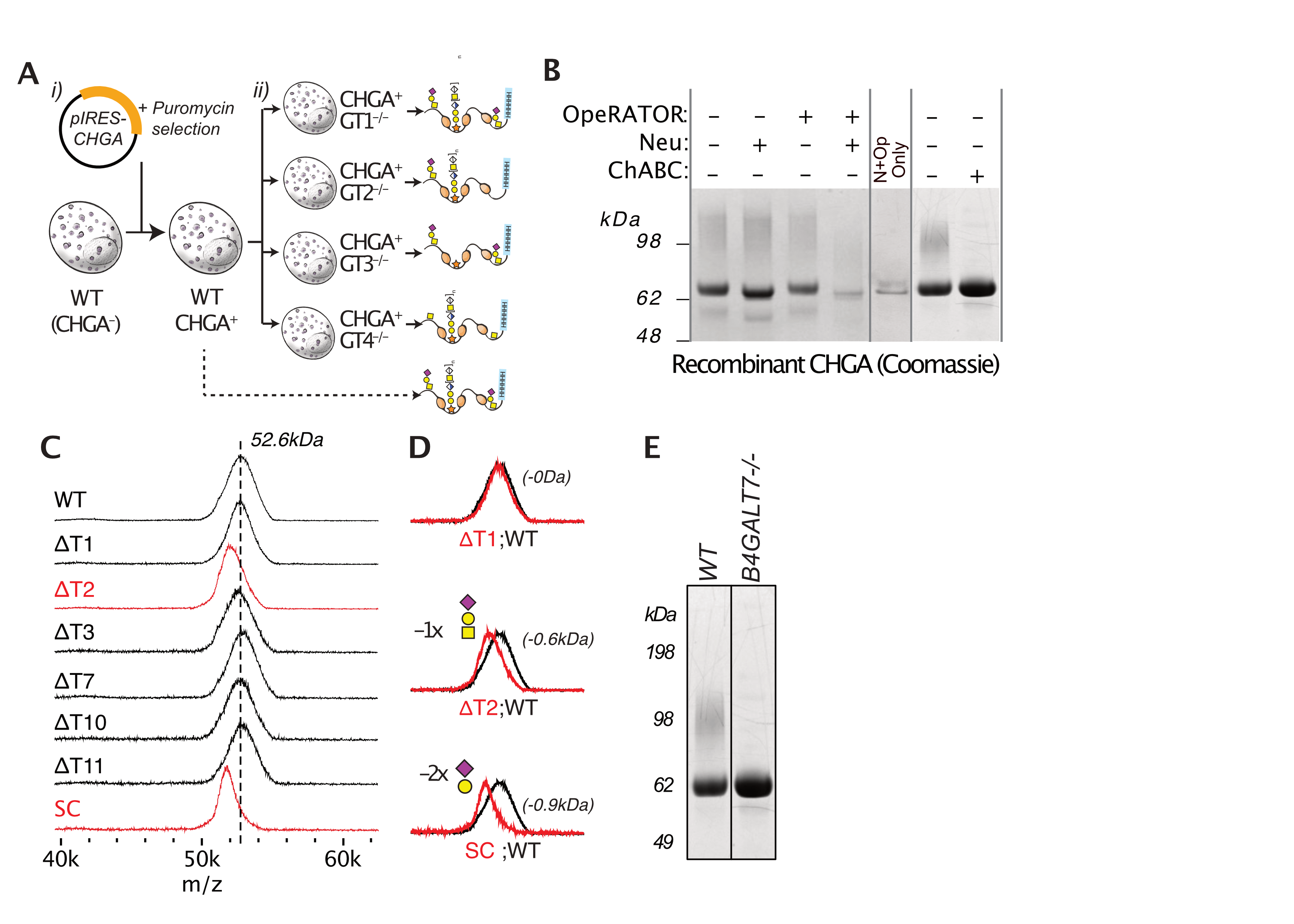


**Supporting figure 6. Recombinant production of CHGA in HEK293.**

**(A**) Schematic of recombinant production of CHGA (TEV-6xHIS tagged) in WT (i) and glycoengineered (ii) cell lines. In brief, HEK293 cells were transfected with CHGA-TEV-6xHIS and selected under puromycin selection. A single clone was isolated and 8 different glycosyltransferases (GT) were individually knocked out using CRISPR-Cas9 gene targeting. Secreted CHGA-TEV-6xHis was purified from conditioned media on NiNTA columns and the C-terminal tags removed by TEV-cleavage. (**B**) SDS-PAGE Coomassie analysis of recombinant CHGA (TEV-HIS tags) produced in HEK293^WT^ pretreated with chABC, OpeRATOR and/or neuraminidase (Neu) as indicated. Note that a minor, slightly faster migrating band remained with HEK293 cell-produced CHGA, which could indicate the presence of a fraction of CHGA fully devoid of O-glycans, however, HEK293 cells also contain some biosynthetic capacity for Core 2 O-glycosylation, which is partly resistant to cleavage of OpeRATOR. N+Op only: Neuraminidase and Operator were separated without the presence of CHGA and migrated at a slightly lower Mw than non-modified CHGA. (**C**) MALDI-TOF analysis of 8 CHGA-TEV-HIS products from WT or glycosyltransferase KO cell lines. The vertical dotted line represents the center of WT m/z. Theoretical non-glycosylated molecular weight of CHGA-TEV-HIS: 51.6 kDa. (**D**) Overlay of mass spectra obtained from CHGA-TEV-HIS produced in ΔT1, ΔT2 and SC cell lines (red) and WT (black). Peak center m/z shift between KO and WT product is given in parentheses. (**E**) Coomassie stain of purified recombinant CHGA produced in HEK293^WT^ or HEK293^ΔB4GALT7^.

**Supporting Figure 7**


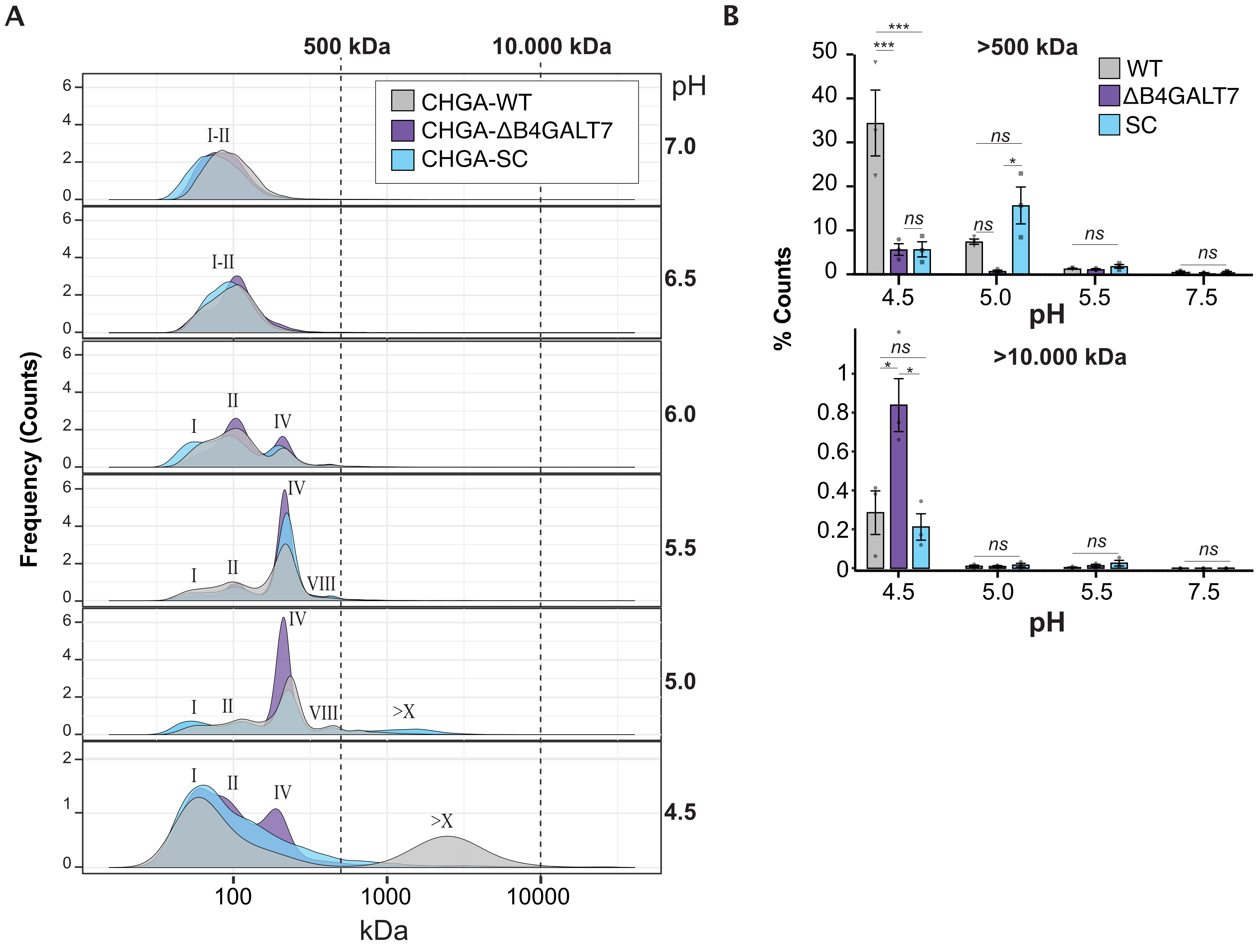


**Supporting figure 7. Mass photometry raw counts**

(**A**) Raw counts from mass photometry analysis of the number of CHGA self-aggregated complexes formed at 200nM concentration at different pH. Roman numbering (I, II, IV, VIII) refers to mono, di, tetra, and octamer homomeric CHGA complexes, while masses >500 kDa (X) correspond to aggregates of >10 CHGA molecules. Recombinant secreted CHGA glycoproteoforms were isolated from HEK293^WT^, HEK293^SC^ and HEK293^ΔB4GALT7^ as indicated. (**B**) Quantification of raw counts at masses >500kDa (top panel) and >10.000kDa (Bottom panel) at different pH values.

**References:**

E. Sjostedt, W. Zhong, L. Fagerberg, M. Karlsson, N. Mitsios, C. Adori, et al., An atlas of the protein-coding genes in the human, pig, and mouse brain, *Science*, **367**, 2020, eaay5947.

C. Steentoft, S.Y. Vakhrushev, H.J. Joshi, Y. Kong, M.B. Vester-Christensen, K.T. Schjoldager, et al., Precision mapping of the human O-GalNAc glycoproteome through SimpleCell technology, *EMBO J.*, **32**, 2013, 1478–1488.
